# Modeling Decadal Organosulfates in Particulate Matter to Improve Estimations of Aerosol Hygroscopicity in the United States

**DOI:** 10.1021/acs.est.6c08752

**Published:** 2026-11-02

**Authors:** Alan R. Gonzalez, Heewon Yim, Kyle P. McCary, Xiangxinyue Meng, Ajith Thenoor Chandran, Yue Zhang, Yuzhou Wang

**Affiliations:** 1 Zachry Department of Civil and Environmental Engineering, 14736 Texas A&M University, College Station, Texas 77843, United States; 2 Department of Atmospheric Sciences, Texas A&M University, College Station, Texas 77843, United States

**Keywords:** Particulate Matter, Organic Aerosol, Isoprene SOA, Chemical Transport Models, Aerosol Hygroscopicity

## Abstract

Organosulfates (OSs) can comprise a substantial fraction of secondary organic aerosol and exhibit significantly different hygroscopicity than inorganic sulfates, yet their spatiotemporal prevalence and climatic impacts remain poorly constrained because speciated measurements are sparse and common chemical transport models do not explicitly represent OS formation. This study develops a flexible and robust modeling framework to quantify isoprene-derived OS (iOS) concentrations and OS impacts on aerosol hygroscopicity (κ) across the contiguous United States (U.S.) for summers spanning 2013–2022. Using the Community Multiscale Air Quality (CMAQ) model with updated iOS chemistry, simulations reproduce Interagency Monitoring of Protected Visual Environments (IMPROVE) network-derived organosulfur and inorganic sulfate and agree with speciated iOS measurements from filter analyses. OS concentrations are elevated in the eastern U.S., with summertime mean values up to 0.51 μg m^–3^. OS formation produces a net 5.6% decrease in κ (direct OS contribution, +2.8%; indirect, −8.4% via sulfate reduction). We apply an interpretable machine-learning surrogate to identify predictive features associated with OS variability and enable efficient estimation without rerunning CMAQ. These results provide the first observation-validated, high-spatial-coverage OS reconstruction and nationwide quantification of OS-driven κ changes, underscoring the importance of representing OS formation and expanded observations to improve aerosol-cloud radiative effect assessments.

## Graphical abstract

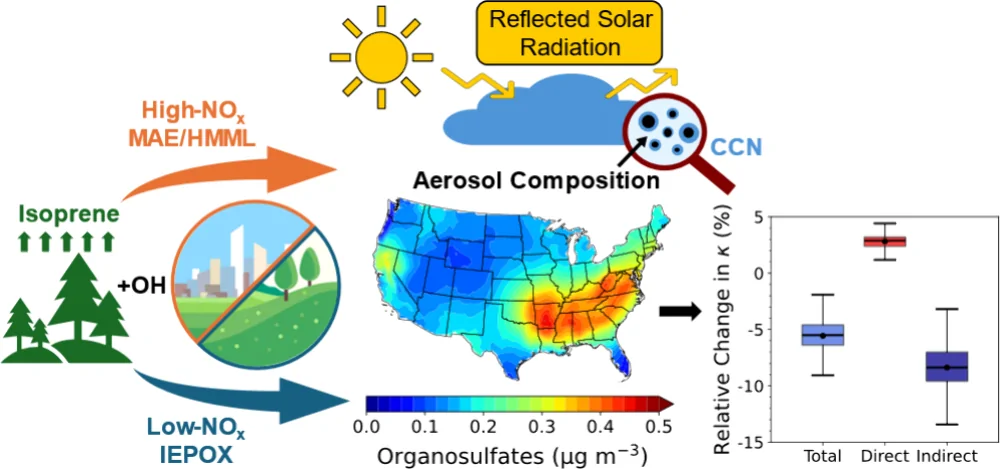

## 1 Introduction

Atmospheric aerosols play an important role in air quality and Earth’s radiative budget.^1,2^ Defined as small particles suspended in air, aerosols can reduce visibility, cause respiratory and cardiovascular disease, and either warm or cool the atmosphere depending on their components.^3,4^ In addition to direct radiative effects through scattering and absorption, aerosols also pose an indirect effect on climate by serving as cloud condensation nuclei (CCN) and ice-nucleating particles (INPs) that modify cloud microphysical properties.^5–8^ Aerosol chemical composition influences particle hygroscopicity and thus its ability to serve as CCN, which is commonly quantified using a single hygroscopicity parameter kappa (κ).^9^ For a given aerosol number-size distribution and supersaturation, higher κ enhances CCN activation and may increase cloud optical thickness and albedo, thereby contributing to a cooling effect.^10,11^ Hygroscopicity also affects aerosol water uptake and particle growth, influencing aerosol optical properties and aerosol optical depth (AOD). Inorganic aerosols tend to show higher κ values while organics have lower κ values, reflecting the high solubility and dissociation of many inorganic salts, whereas κ for secondary organic aerosols (SOAs) can be strongly influenced by molecular weight.^12–14^

Atmospheric aerosols originate from both primary emissions and secondary formation, with major secondary components including sulfates, nitrates, ammonium, and SOAs. SOAs form when volatile organic compounds (VOCs) from either biogenic or anthropogenic sources are oxidized in the atmosphere, and they represent a substantial fraction of organic aerosol (OA) mass.^15–18^ Among SOA constituents, organosulfates (OSs), organic compounds characterized by sulfate ester functional groups, can contribute up to 30% of total OA mass.^19–22^ OS concentrations are often higher in certain regions of the United States (U.S.), such as the Southeast U.S., where abundant biogenic and anthropogenic emissions coincide with warm and humid meteorological conditions that promote OS formation.^23–26^ However, due to large uncertainties in emissions and complex multiphase reaction pathways, quantifying SOA concentrations, especially OSs, remains challenging and represents an important gap in atmospheric research.^27–29^ Moreover, because OS formation is not explicitly represented in many default chemical transport model (CTM) simulations, OS mass can be underestimated or not simulated. Therefore, the modeled allocation of sulfur between inorganic sulfate and organic sulfur-containing aerosol components may be biased. This bias can propagate to estimates of aerosol composition and aerosol hygroscopicity, and consequently to associated aerosol-cloud interactions and radiative effects.

Isoprene-derived OSs (iOSs) are among the best characterized OS species. They form via multiphase reactions of isoprene oxidation products on acidic sulfate aerosols under distinct low- and high-NO_*x*_ pathways,^30,31^ and have been directly quantified through both airborne measurements and chemical analysis of fine particulate matter (PM_2.5_) filter samples at a limited number of sites.^25,32–34^ iOSs can also be derived from CTM simulations, whereas other OS species remain largely unknown or unquantified in both measurements and modeling frameworks. Previous studies suggest that iOSs contribute ∼30% of the total organosulfur (OrgS) mass in the eastern U.S.^35^ Here, OSs are the broader climate-relevant compound class of interest, iOSs are the isoprene-derived subset of OSs that can be chemically measured or estimated by modeling, and OrgS is a sulfur-equivalent quantity representing sulfur contained in OSs and other organosulfur compounds.

Unlike speciated OSs or iOSs, for which measurements are sparse in space and time, OrgS can be indirectly inferred from routine national monitoring networks, providing a broader observational constraint on organosulfur abundance. For example, 24 h filter samples collected every 3 days by the Interagency Monitoring of Protected Visual Environments (IMPROVE) network provide total particulate sulfur measured by X-ray fluorescence (XRF) and inorganic sulfate measured through ion chromatography (IC).^36^ If particulate sulfur were dominated by inorganic sulfate alone, total particulate sulfur from XRF and inorganic sulfate-derived sulfur from IC would be expected to agree within measurement uncertainty. However, studies have reported significant differences between these two quantities, indicating the presence of organosulfur compounds and associated OrgS.^37,38^ Targeted analyses indicate that iOSs explain certain amounts of the inferred OrgS, or “missing sulfur”, implied by IMPROVE sulfur measurements, while other organosulfur species and formation pathways that contribute to OrgS remain poorly characterized.^35^ Despite recent advances, OS quantification remains limited in spatial and temporal coverage, leaving large gaps in their prevalence and variability, as well as climate-relevant impacts.

In this study, a flexible and comprehensive framework is developed to estimate OS concentrations and assess their climatic impacts across the U.S. Key precursors and formation pathways for iOSs are simulated using the Community Multiscale Air Quality (CMAQ) model over a 10-year span (2013–2022). iOS concentrations are modeled using CMAQ, and OS and OrgS concentrations are then derived through additional postprocessing calculations. The derived OrgS and modeled inorganic sulfate are evaluated against IMPROVE measurements. We then quantify the impact of OSs on aerosol hygroscopicity and apply machine-learning techniques to identify the primary predictive features of OS formation. Overall, this work (1) provides the first long-term, nationwide modeling assessment of OS prevalence in the United States, (2) quantifies how OS-driven changes in aerosol composition affect key climate-relevant factors, and (3) offers a generalizable methodology framework for OS quantification with broad spatiotemporal coverage.

## 2 Materials and Methods

### 2.1 Modeling and Analytical Framework

Figure 1 summarizes our modeling and analytical framework. We employed CMAQ with updated isoprene chemistry to simulate iOS concentrations, using meteorological fields and emissions as key inputs. We evaluated model performance against ground-based measurements from the IMPROVE network. To isolate the impacts of OS on aerosol hygroscopicity, we calculated κ from two simulations: (1) the baseline model without OS formation and (2) the updated model including OS formation. Comparing these cases quantified the direct influence of OS on κ, as well as indirect effects mediated through OS-driven changes in other aerosol species (e.g., inorganic sulfate). Finally, we applied machine-learning methods to predict and attribute OS variability to key meteorological, emissions, and chemical features, providing both a relative-importance assessment and an empirical approach for OS estimation.

**Figure 1:**
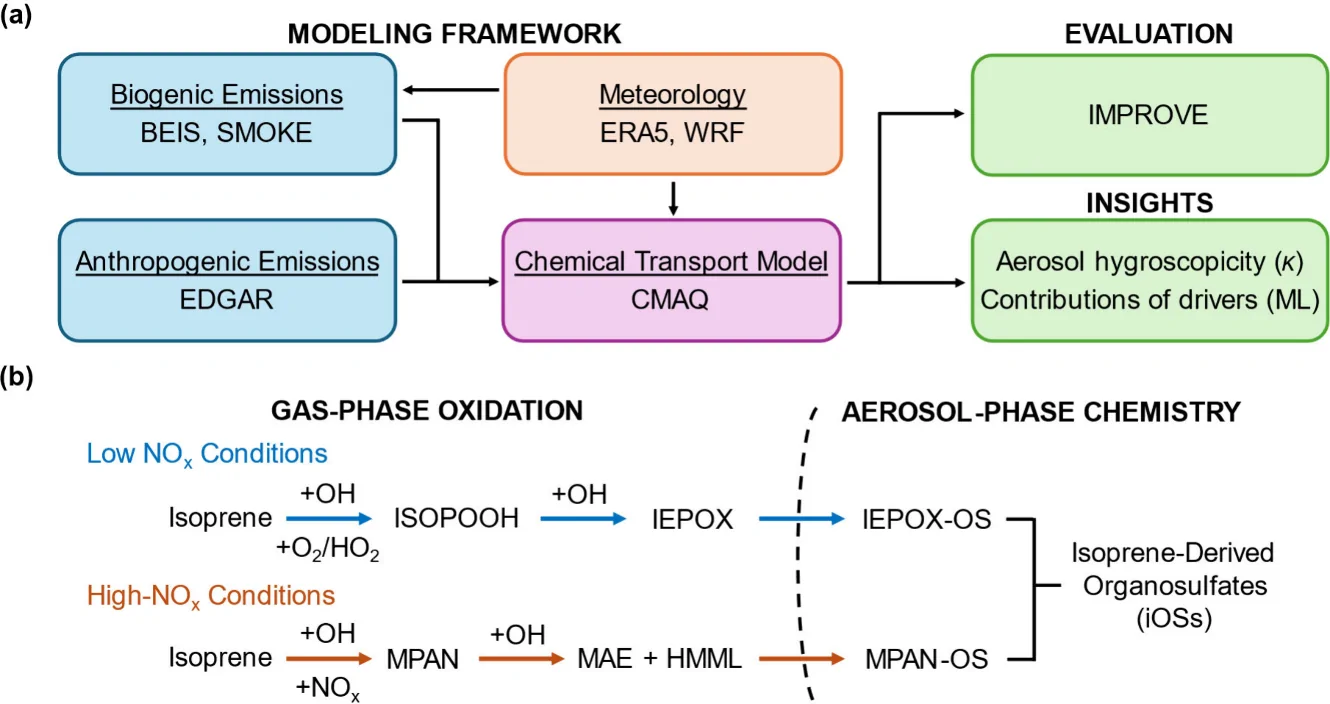
(a) Modeling framework and (b) reaction mechanisms for the formation of isoprene-derived organosulfates. Intermediate products include isoprene hydroxy hydroperoxides (ISOPOOH), isoprene epoxydiols (IEPOX), methacryloyl­peroxynitrate (MPAN), methacrylic acid epoxide (MAE), and hydroxymethyl-methyl-α-lactone (HMML).

### 2.2 Model Domain and Meteorological Data

Model simulations for this study were conducted for a domain covering the contiguous U.S. at a horizontal grid resolution of 36 km × 36 km. The study period included the summer months (June, July, August; JJA) of 2013, 2016, 2019, and 2022. For analysis of output concentrations, geographic regions within the U.S. (Figure S1) were defined based on the 10 regional offices of the Environmental Protection Agency (EPA).

As shown in Figure 1a, meteorological initial and boundary conditions on surface and pressure levels were obtained from ERA5, the fifth-generation reanalysis data set of global climate produced by the European Centre for Medium-Range Weather Forecasts (ECMWF).^39,40^ The input data were processed using the Weather Research and Forecasting (WRF) model^41^ to provide hourly meteorological conditions for the model domain on 44 vertical levels.

### 2.3 Biogenic and Anthropogenic Emissions

Biogenic emissions were processed offline using the meteorological data as input and the Biogenic Emission Inventory System (BEIS) version 3.7 built within the Sparse Matrix Operator Kernel Emissions (SMOKE) system version 4.8.1.^42^ Gridded land use data were based on the Biogenic Emissions Landcover Database version 5 (BELD5). With the benefits of global coverage and monthly resolution, anthropogenic emissions were obtained from the Emissions Database for Global Atmospheric Research (EDGAR) database at 0.1° × 0.1° resolution.^43^ Emission sectors included power industry, industrial combustion, buildings, transport, agriculture, fuel exploitation, industrial processes, and waste.

An in-house set of programs was used to prepare and match the spatial and temporal resolutions of the anthropogenic emissions to the model domain (Figure S2). Sector-specific temporal and vertical profiles were applied to produce hourly, vertically resolved anthropogenic emissions of SO_2_, CO, and NH_3_, along with speciated NO_*x*_, PM_2.5_, and VOC emissions.^44,45^ As a sensitivity test of emission-inventory choice, additional model simulations for July 2016 were completed using anthropogenic emissions from the EPA 2016 National Emission Inventory (NEI) platform.

### 2.4 iOS Simulation and Scaling

We used CMAQ version 5.0.1 ^46^ with the sixth-generation aerosol module (AERO6) to quantify the iOS concentrations. Model inputs included meteorological data, biogenic emissions, and anthropogenic emissions (Figure 1a). CMAQ used the SAPRC-11 gas-phase chemical mechanism with additional isoprene oxidation chemistry and represented isoprene-derived SOA formation through reactive aerosol uptake processes.^47,48^ Figure 1b illustrates the low- and high-NO_*x*_ pathways corresponding to modeled iOSs derived from isoprene epoxydiols (IEPOX) and methacryloyl­peroxynitrate (MPAN), represented as IEPOX-OS and MPAN-OS, respectively. These parametrizations were constrained by laboratory and field-campaign observations, including measurements from the 2013 Southern Oxidant and Aerosol Study (SOAS), and provide the mechanistic basis for simulating iOS formation through coupled gas-phase oxidation, aerosol uptake, transport, and loss processes.^49,50^ Raw modeled iOS was therefore defined as the sum of IEPOX-OS and MPAN-OS. Additional details on the CMAQ modeling scheme and underlying physicochemical mechanisms are provided in Supporting Information, Text S1.

Previous CMAQ applications have shown that the raw model predictions agree with isoprene-derived aerosol tracer measurements from field campaigns on selected sites over specific time periods, including 2013 SOAS.^23,24,50^ Because the national evaluation in this study relies on the IMPROVE network, we used the chemically characterized 2016 IMPROVE PM_2.5_ filter samples from Chen et al.^35^ as an IMPROVE-based constraint on modeled iOS concentrations. Although iOSs are not measured routinely by IMPROVE, this data set provides chemically characterized iOS measurements from 17 sites across the contiguous U.S. Measurements from SOAS and the IMPROVE filter samples both indicate that methyltetrol sulfate and 2-methylglyceric acid sulfate (denoted in this study as IEPOX-OS and MPAN-OS, respectively) are dominant measured iOS components. However, the two data sets differ in both magnitude and relative contribution. Specifically, the average IEPOX-OS + MPAN-OS concentration at Centreville, AL during 2013 SOAS was substantially higher than the maximum value observed across the chemically characterized 2016 IMPROVE sites, 676 versus 140 ng m^–3^, respectively.^23,35^ IEPOX-OS + MPAN-OS also accounted for a larger fraction of total iOS at Centreville during SOAS than across the IMPROVE sites, 82% versus approximately 60%. Elevated concentrations of IEPOX-OS + MPAN-OS have also been reported in urban or near-source environments, including 1845 ng m^–3^ in an Atlanta, GA study in 2015.^25^ These differences may reflect differences in site environment, sampling design, analytical methods, meteorology, precursor emissions, and the regional/background nature of the IMPROVE network.

Given the magnitude difference between the SOAS and IMPROVE-based iOS measurements, we applied a postprocessing adjustment factor to the raw CMAQ-predicted IEPOX-OS + MPAN-OS concentrations. The main analysis used a uniform adjustment factor of 0.2 (eq 1), derived from the ratio between the maximum IEPOX-OS + MPAN-OS concentration reported at a Southeast IMPROVE site near the SOAS region and the average concentration measured during SOAS at Centreville, AL:

1 $${\mathrm{iOS}}_{\mathrm{model}}=\left(0.2\right)\left(\left[\mathrm{IEPOX‐OS}\right]+\left[\mathrm{MPAN‐OS}\right]\right)$$

where [IEPOX-OS] and [MPAN-OS] are the raw CMAQ-predicted iOS concentrations. This uniform factor was used as the base case because it provides a measurement-derived scale adjustment for the overall SOAS–IMPROVE magnitude difference while preserving the spatial and temporal variability simulated by CMAQ.

To evaluate sensitivity to this adjustment-factor assumption, we considered four additional cases, including two uniform adjustment factors and two location-based adjustment factors with lower- and upper-bound values, respectively (see Supporting Information, Text S2 and Table S1). The location-based factors were derived from site-specific ratios between chemically characterized 2016 IMPROVE iOS measurements and corresponding raw CMAQ-predicted iOS concentrations, then interpolated across the model domain (Figures S3a and S4a). These location-based cases allow both the magnitude and spatial pattern of the adjustment to vary, but they also incorporate uncertainties from both sides of the model–measurement comparison and may be sensitive to local model–measurement mismatch when applied across the domain. We therefore used the uniform adjustment factor as the base case and treated the location-based adjustment factors as complementary sensitivity tests. Modeled OrgS concentrations and subsequent κ calculations were evaluated for the base case and all four sensitivity cases.

### 2.5 Derivation of OrgS and OS from Modeled iOS

To support model evaluation and enable subsequent κ calculations, we used location-dependent scaling factors to map iOS_model_ to total particulate OrgS and corresponding OS mass. The scaling was based on the fractions of isoprene-derived OrgS to total OrgS (iOrgS/OrgS) derived from chemically resolved IMPROVE filter analyses by Chen et al.^35^ In their study, reported iOrgS/OrgS fractions ranged from 0.01 to 0.37, with an average of 0.17. We generated a gridded iOrgS/OrgS field at the 36 km × 36 km resolution (Figure S5) using linear radial basis function interpolation based on distances to all measured sites. Because very small iOrgS/OrgS fractions can substantially increase the scaled total OrgS and OS estimates, we imposed a lower bound of 0.17, corresponding to the mean observed fraction across the contiguous U.S. To evaluate sensitivity to this iOrgS/OrgS scaling assumption, we conducted three additional tests: (1) a uniform iOrgS/OrgS value of 0.17 across all grids, (2) the gridded iOrgS/OrgS field with no lower bound, and (3) the gridded field with an alternative lower bound of 0.1 (Figure S6).

For evaluation with IMPROVE measurements, total OrgS from the modeled results (OrgS_model_) was calculated by converting the modeled concentrations of iOSs to sulfur mass and scaling by iOrgS/OrgS (eq 2):

2 $${\mathrm{OrgS}}_{\mathrm{model}}=\frac{\left(0.2\right)\left(\frac{32}{216}\left[\mathrm{IEPOX‐OS}\right]+\frac{32}{200}\left[\mathrm{MPAN‐OS}\right]\right)}{\mathrm{iOrg}S/\mathrm{OrgS}}$$

where the molecular-weight ratios convert the aerosol-phase mass concentrations of the modeled iOSs to their corresponding sulfur mass. The numerators are the atomic weight of sulfur (32 g mol^–1^), and the denominators are the molecular weights of the iOSs.^51^

To represent the broader climate-relevant compounds of interest, corresponding total OS mass concentrations (OS_model_) were calculated by scaling iOS_model_ by iOrgS/OrgS (eq 3):

3 $${OS}_{model}=\frac{{iOS}_{model}}{iOrgS/OrgS}$$

In addition to OS and OrgS, the comprehensive set of CMAQ output gas and particle-phase species were also considered for model evaluation, estimation of aerosol hygroscopicity, and decomposition of predictive features.

### 2.6 Evaluation with Measurements

Direct, nationwide evaluation of OS is limited by sparse, speciated OS observations. We therefore prioritized evaluation using IMPROVE, which provides long-term, spatially extensive PM_2.5_ composition measurements and enables an indirect constraint on particulate OrgS. Specifically, model-derived OrgS was evaluated primarily against OrgS inferred from routine IMPROVE measurements from 2013 to 2022 of total particulate sulfur and inorganic sulfate. Inorganic sulfate was evaluated in parallel to ensure that the sulfate aerosol system that underpins the OS formation was robustly represented.

To obtain a comprehensive spatial and temporal evaluation of our modeled OrgS, concentrations of total particulate sulfur by XRF and inorganic sulfate by IC were retrieved for a total of 15 906 site-day observations across 152 IMPROVE sites in the contiguous U.S.^36^ As shown in eq 4, concentrations of OrgS from the IMPROVE sites (OrgS_IMPROVE_) were determined based on the measured XRF total sulfur ([S]_XRF_) and IC inorganic sulfate ([SO_4_]_IC_):

4 $${OrgS}_{IMPROVE}={\left[S\right]}_{XRF}-\frac{32{\left[SO_{4}\right]}_{IC}}{96}$$

where the atomic/molecular weights of sulfur and sulfate are 32 and 96 g mol^–1^, respectively. Only measurements that exceeded the sample-specific minimum detection limits (∼0.3 ng m^–3^ and ∼10 ng m^–3^ for [S]_XRF_ and [SO_4_]_IC_, respectively) and yielded positive estimates of OrgS were retained.

Model performance was evaluated using the slope of linear regression, coefficient of determination (*R* ^2^), and Spearman’s rank correlation coefficient (ρ). Model–observation comparisons were performed for both OrgS and inorganic sulfate and were assessed for both spatial patterns (monthly mean concentrations in each grid cell) and temporal variability (average daily concentrations). We conducted these evaluations for the contiguous U.S. and separately for each EPA Region.

In addition to this indirect evaluation, we directly evaluated modeled iOS concentrations by comparing iOS_model_ at corresponding IMPROVE sites and sampling dates to speciated iOS measurements. These measurements were obtained from chemically characterized IMPROVE filters collected in July and August 2016.^35^

### 2.7 Estimations of Aerosol Hygroscopicity

Aerosol hygroscopicity for the modeled results (κ_model_) was estimated based on the particle chemical composition using the Zdanovskii–Stokes–Robinson (ZSR) mixing rule, as described in Petters and Kreidenweis.^9^ The composition-based implementation followed the procedure described by Huang et al.^52^ Under internal mixing conditions, total κ is calculated using eq 5:

5 $$\kappa_{model}=\underset{i}{\sum }\left(\varepsilon_{i}\kappa_{i}\right)$$

where ε_*i*_ and κ_*i*_ are the volume fraction and hygroscopicity parameter for the individual (dry) components. Neutral inorganic salts (NH_4_NO_3_, H_2_SO_4_, NH_4_HSO_4_, and (NH_4_)_2_SO_4_), organics, inorganic sea salt, black carbon, and OSs were considered as individual components of the total κ. For all κ calculations, the OS component was represented by postprocessed OS_model_ concentrations, which incorporated the corresponding iOS adjustment factor and iOrgS/OrgS scaling factor. For OSs, we used κ_OS_ = 0.139.^53^ As a sensitivity test, we also assigned OSs the lumped organics value (κ_OS_ = κ_org_ = 0.1) and quantified the resulting change in the total κ. Following Huang et al.,^52^ the κ for black carbon was set to zero. The volume fractions for each component were determined by dividing their mass concentrations by their densities. Since CMAQ output provides the particle-phase concentrations of SO_4_^2–^, NH_4_^+^, and NO_3_^–^, the ion pairing scheme described by Gysel et al.^54^ was used to reconstruct the corresponding concentrations of the neutral inorganic salts for the κ calculation. The particle mass concentrations of Na^+^ and Cl^–^ were added to represent inorganic sea salt.

To evaluate the impacts of OSs on κ, we compared κ_base_ (baseline case without OS formation) with κ_w/OS_ (OS-including case), both computed using eq 5. The direct OS impact was defined as the OS contribution (ε_OS_κ_OS_) to bulk κ in the OS-including case, where ε_OS_ is the OS volume fraction in the OS-including case. The indirect effect represented OS-driven changes in the remaining aerosol components, dominated by changes in inorganic sulfate due to OS formation. The total, direct, and indirect changes were reported as relative changes using eqs 6–8:

6 $${\Delta \kappa }_{Total}=\frac{\kappa_{w/OS}-\kappa_{base}}{\kappa_{base}}\times 100\%$$

7 $${\Delta \kappa }_{Direct}=\frac{\varepsilon_{OS}\kappa_{OS}}{\kappa_{base}}\times 100\%$$

8 $${\Delta \kappa }_{Indirect}={\Delta \kappa }_{Total}-{\Delta \kappa }_{Direct}$$

where Δκ (total, direct, and indirect) is the change in hygroscopicity (κ) with reference to the base case. To further assess aerosol-cloud implications, supplemental CCN calculations were performed using κ_base_, κ_w/OS_, and modeled fine-mode aerosol number-size distributions at selected supersaturations; methodological details are provided in Supporting Information, Text S3.

### 2.8 Decomposition of Primary Predictive Features

To identify and provide potential insights on the primary meteorological and gas-phase predictive features associated with modeled OS variability, an Extreme Gradient Boosting regression model (XGBoost)^55^ with a squared error objective function was applied. The XGBoost model was selected due to its ability to capture the nonlinear nature of atmospheric chemistry and multicollinearity between features. Model interpretability was assessed using SHapley Additive exPlanations (SHAP), which quantify feature importance and the direction of each feature’s contribution to model predictions.^56,57^ Since SHAP values describe statistical attribution, they were interpreted as predictive associations rather than causal effects. After preliminary screening of closely related and partially redundant variables (e.g., included relative humidity but not water vapor mixing ratio), the meteorological and gas-phase features in the XGBoost model with the highest mean absolute SHAP values were identified as key predictive features that represented atmospheric conditions and adequately predicted concentrations of OSs.

For each year and grid cell in the model domain, monthly averages of the predictor and outcome variables were calculated and randomly split into the training and testing sets. A fixed random seed was used to ensure reproducibility, and tuning of hyperparameters included using grid search with 3-fold cross-validation. While minimizing the mean-squared error, the best performing model was applied to the test set, and the corresponding root-mean-squared error (RMSE) and *R* ^2^ values were determined. As a robustness check, we trained random forest and ridge regression models using the same predictors and compared their performance and feature-importance rankings.

## 3 Results and Discussion

### 3.1 Modeled Concentrations of Organosulfates

Monthly mean concentrations of iOS_model_ demonstrated persistent enhancement in the southeastern U.S. (Figure S7). As shown in Figure S8, national mean IEPOX-OS and MPAN-OS particulate concentrations from the CMAQ model output across all years were 240 ng m^–3^ and 4 ng m^–3^, respectively. IEPOX-OS concentrations were 1–2 orders of magnitude higher than MPAN-OS concentrations, indicating that the IEPOX pathway (low NO_*x*_ conditions) dominated iOS formation (∼98%).

Figure 2 shows the spatial and temporal patterns of summertime average predictions of OS concentrations for 2013, 2016, 2019, and 2022. Spatially, summertime OS levels were higher in the eastern U.S. than in the western and central U.S., although the contrast was less extreme than for iOSs because scaling to total OSs incorporated spatial variability in the iOrgS/OrgS ratio (Figure S5), which was higher in the eastern U.S. Summertime OS concentrations generally decreased across the eastern U.S. from 2013 to 2022; however, the nationwide mean in 2022 (0.20 μg m^–3^) was higher than several prior years. The national average for all simulated years indicated that summertime OS concentrations accounted for 10% of total organic aerosols. Across months (Figure S9), OS concentrations were highest in July, with a multiyear national average of 0.22 μg m^–3^. An exception to this pattern occurred in August 2013, which had the highest national average of 0.27 μg m^–3^.

**Figure 2:**
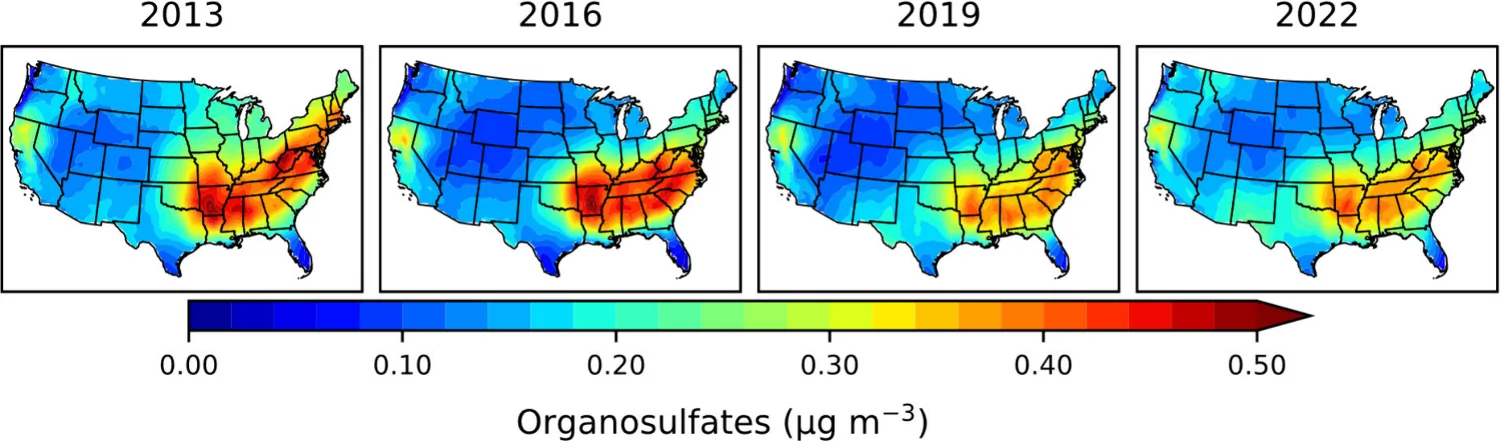
Average summertime (JJA) concentrations of modeled organosulfates.

Summaries on EPA regions (Figure S10) further show the highest mean OS concentrations in the Mid-Atlantic (Philadelphia office; 0.35 μg m^–3^) and Southeast (Atlanta; 0.32 μg m^–3^) regions, where peak values exceeded 0.40 μg m^–3^. The South Central (Dallas) and Midwest (Kansas City) had lower regional means (∼0.22 μg m^–3^) but comparable peak values, while the northeastern (Boston and New York) regions exhibited intermediate concentrations. OS concentrations were relatively lower in the remaining regions, although localized hotspots, such as northern California, could exceed 0.25 μg m^–3^.

### 3.2 Evaluation with Site-Based Measurements

Evaluation included spatial and temporal comparisons of OrgS_model_ concentrations with measurements from IMPROVE sites. Across all summer months, both the model and observations consistently showed higher OrgS in the eastern U.S. than in the western regions (Figures 3a and S11). In the Southeast region, the model reproduced the observed day-to-day variability, including the peaks and troughs of the OrgS time series (Figure 3b). When comparing site-level monthly means (Figure 3c), the model slightly underestimated OrgS concentrations at higher levels and showed a modest low bias (linear regression slope = 0.80) but maintained a strong overall agreement (*R* ^2^ = 0.82; Spearman ρ = 0.59). Sensitivity tests using alternative iOS_model_ adjustment factors showed that the absolute OrgS magnitude was sensitive to the adjustment-factor assumptions (Table S1). The uniform lower- and upper-bound cases shifted the regression slopes to 0.64 and 1.40, respectively, and maintained the *R* ^2^ value; the location-based cases showed lower agreement, with slopes of 0.36–0.68 and *R* ^2^ values of 0.73–0.78 (Figures S3 and S4). These results further support using the uniform 0.2 factor as the base case because it avoids the stronger under- or overestimation observed in the sensitivity cases.

**Figure 3:**
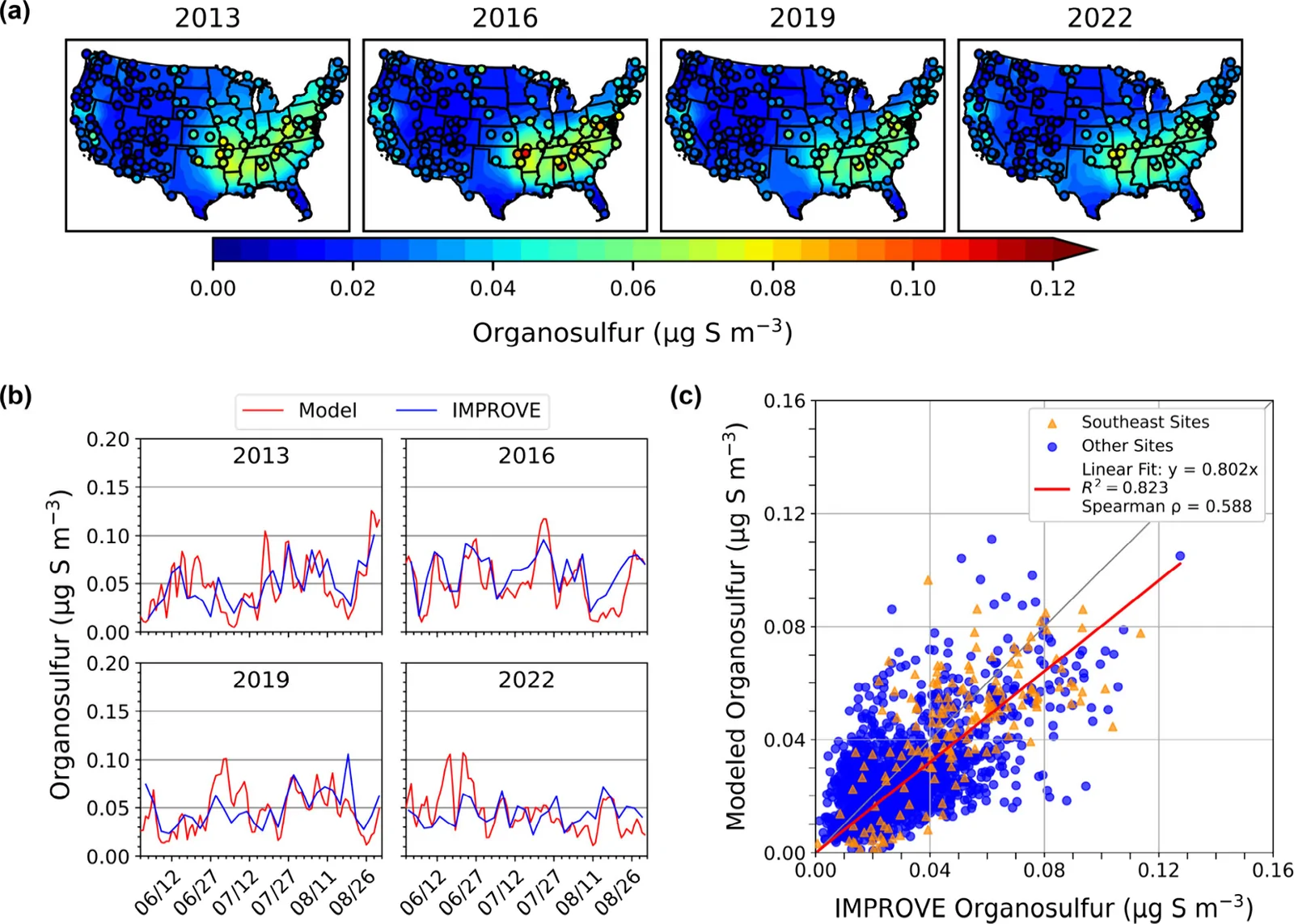
Evaluation of modeled organosulfur concentrations with IMPROVE site data. Evaluation includes (a) maps of average summertime concentrations, (b) time series comparisons of average concentrations in the Southeast region, and (c) a scatter plot of monthly average concentrations per site.

Similar conclusions were evident when assessing each region separately, with an *R* ^2^ value range of 0.82–0.91 in the eastern and central regions, and slight underestimation and relatively lower *R* ^2^ values (0.69–0.75) in the western regions (Figures S12 and S13). The model exhibited an average bias of approximately ±0.05 μg S m^–3^ and a mean fractional bias (MFB) of −17% across all years when compared to spatially interpolated IMPROVE site data (Figures S14 and S15). The model slightly underestimated OrgS concentrations in the Midwest and southern California and overestimated them in northern California, with no significant systematic biases in other regions. As shown in the sensitivity tests (Figure S16), estimated OrgS concentrations are highly sensitive to the iOrgS/OrgS scaling factors, indicating that uncertainty and potential bias in the scaling factors in this region were a major contributor to the overestimation in northern California. Applying a minimum threshold of 0.17 to the location-dependent scaling factors reduced the bias compared with cases using lower or no thresholds.

Further supporting model robustness, modeled inorganic sulfate concentrations showed strong spatial and temporal agreement with IMPROVE observations (Figures S17–S19). In the site-level spatial comparison, the OS-inclusive simulation yielded a linear regression slope of 1.06 and an *R* ^2^ value of 0.87 across all sites, with regional *R* ^2^ values ranging from 0.88 to 0.98. In comparison, the base simulation without OS formation achieved a similar *R* ^2^ value of 0.88 but a slightly higher regression slope of 1.15, indicating a larger positive bias. In the Southeast temporal comparison, inorganic sulfate was overestimated in the base simulation, especially in 2022; including OS formation slightly reduced this positive bias, with model–observation slopes shifting from 1.07–1.71 to 0.99–1.57 across years while *R* ^2^ values remained similar at 0.95–0.98 (Figure S18b). Overall, including OS formation modestly reduced the positive bias in inorganic sulfate concentrations while leaving the representation of spatial and temporal variability largely unchanged.

For additional direct evaluation, we compared our modeled iOS concentrations (Figure S7) with available measurements from IMPROVE sites. For selected sites and dates in July/August 2016, IMPROVE filter samples chemically analyzed by Chen et al.^35^ resulted in an average total iOS concentration of 69.2 ng m^–3^. Across the same sites and dates, our modeled results demonstrated strong agreement, yielding an average iOS_model_ concentration of 71.4 ng m^–3^ and only a slight overestimation of 3% (Table S2). Shown in both the modeled results and site-based data, concentrations of iOSs were lower than 50 ng m^–3^ in western regions and could exceed 175 ng m^–3^ in eastern regions.

As a sensitivity test of anthropogenic emissions, we conducted an additional simulation for July 2016 using the NEI emissions. Predicted OS concentrations were slightly lower but exhibited highly consistent spatial patterns with EDGAR-based simulations (Figures S20 and S21), with a model–model regression slope of 0.74 and an *R* ^2^ value of 0.96. Both simulations showed elevated OS concentrations in the Mid-Atlantic and Southeast, although the NEI case produced lower peak values.

### 3.3 Impacts of Organosulfates on Aerosol Hygroscopicity

Summertime total κ values, which indicated aerosol hygroscopicity under the internal mixing assumption and included the effects of OSs and other aerosol species (e.g., inorganic salts and organics), averaged 0.25 across the contiguous U.S., with lowest values (∼0.2) in the eastern U.S. and higher values (∼0.4) in the West Coast, Gulf Coast, and southern Texas (Figure 4a). Monthly means showed a consistent spatial pattern and similar magnitudes (Figure S22), with slightly lower κ in the Southeast region during July-August 2019. OSs generally contributed to ∼2–6% of total κ across the U.S., with smaller contributions in the coastal regions, and total organics (including OSs) contributed to ∼40% of total κ in the Southeast region and ∼25% elsewhere (Figure S23).

**Figure 4:**
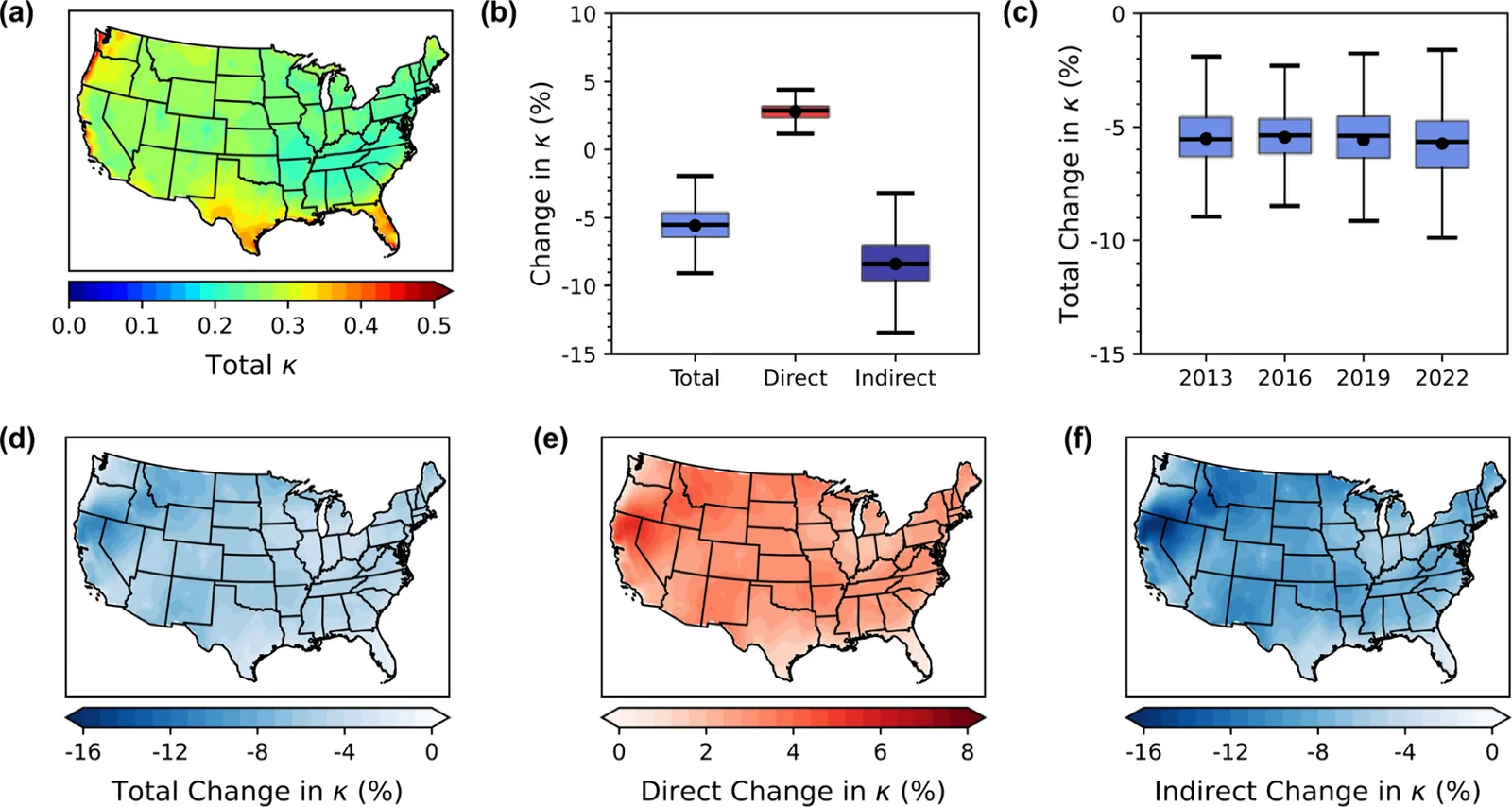
Estimated values for summertime aerosol hygroscopicity based on modeled chemical composition. Data include (a) total κ with organosulfates included, (b) average changes in κ, (c) total change in κ based on year, and (d–f) maps of the average changes in κ. The baseline κ for calculating relative change does not include organosulfates. Direct change indicates adding organosulfates as an individual component in the calculation of κ. Indirect change reflects how κ is impacted by the reduction of inorganic sulfates due to the formation of organosulfates.

We further isolated the total, direct, and indirect impacts of OS formation on total κ by forming comparisons against a base case that did not include OS formation for the summertime modeled results (Figure 4b). On average, the net impact of OSs decreased summertime total κ by 5.6% (Figures 4c,d and S24). Including the direct contribution of OSs in the calculation of total κ yielded an increase of 2.8% (Figure 4e). Meanwhile, OSs indirectly decreased total κ by 8.4% due to the reduction of inorganic sulfate concentrations (Figure 4f). As sensitivity tests, total κ was calculated using the same individual κ value for OSs as bulk organics (0.1). Using an OS-specific κ of 0.139 increased total κ by up to 2% (Figure S25). In addition, sensitivity tests using alternative iOS_model_ adjustment factors consistently showed a net decrease in the total κ when OS formation was included, ranging from 2.4% to 9.2% (Table S1), supporting the robustness of the direction of OS effect on total κ. Consistent with this decrease in κ, including OS formation decreased fine-mode CCN concentrations, with domain-average decreases of up to 6.2% and larger relative decreases under lower-supersaturation conditions (see details in Supporting Information, Text S3 and Figure S26).

### 3.4 Identification of Primary Predictive Features Using Explainable Machine Learning

The formation of OSs is dependent on meteorological conditions and interactions with atmospheric constituents. To identify the dominant predictive features of modeled OS variability, a subset of meteorological variables and precursors were used to train an XGBoost model with 3-fold cross validation. We used SHAP analysis to quantify feature importance and the direction of each feature’s associations with OS concentrations (Figure 5a). Isoprene concentration, near-surface temperature, SO_2_ concentration, and relative humidity demonstrated the highest feature importance based on their mean absolute SHAP values, together accounting for 74.6% of the total SHAP importance. SHAP dependence patterns indicated positive associations of OSs with isoprene, temperature, and SO_2_, whereas relative humidity exhibited a mixed influence (Figures S27 and S28). Additional contributing factors included near-surface wind speed, planetary boundary layer height, and NH_3_, OH, and NO_*x*_ concentrations. Evaluating the optimized model with the test data set yielded an RMSE of 0.04 and an *R* ^2^ value of 0.87. While the XGBoost model demonstrated the best performance, random forest (RMSE = 0.05 and *R* ^2^ = 0.77; Figure S29) and ridge regression (RMSE = 0.08 and *R* ^2^ = 0.56; Figure S30) models yielded consistent feature-importance rankings and directions of influence.

**Figure 5:**
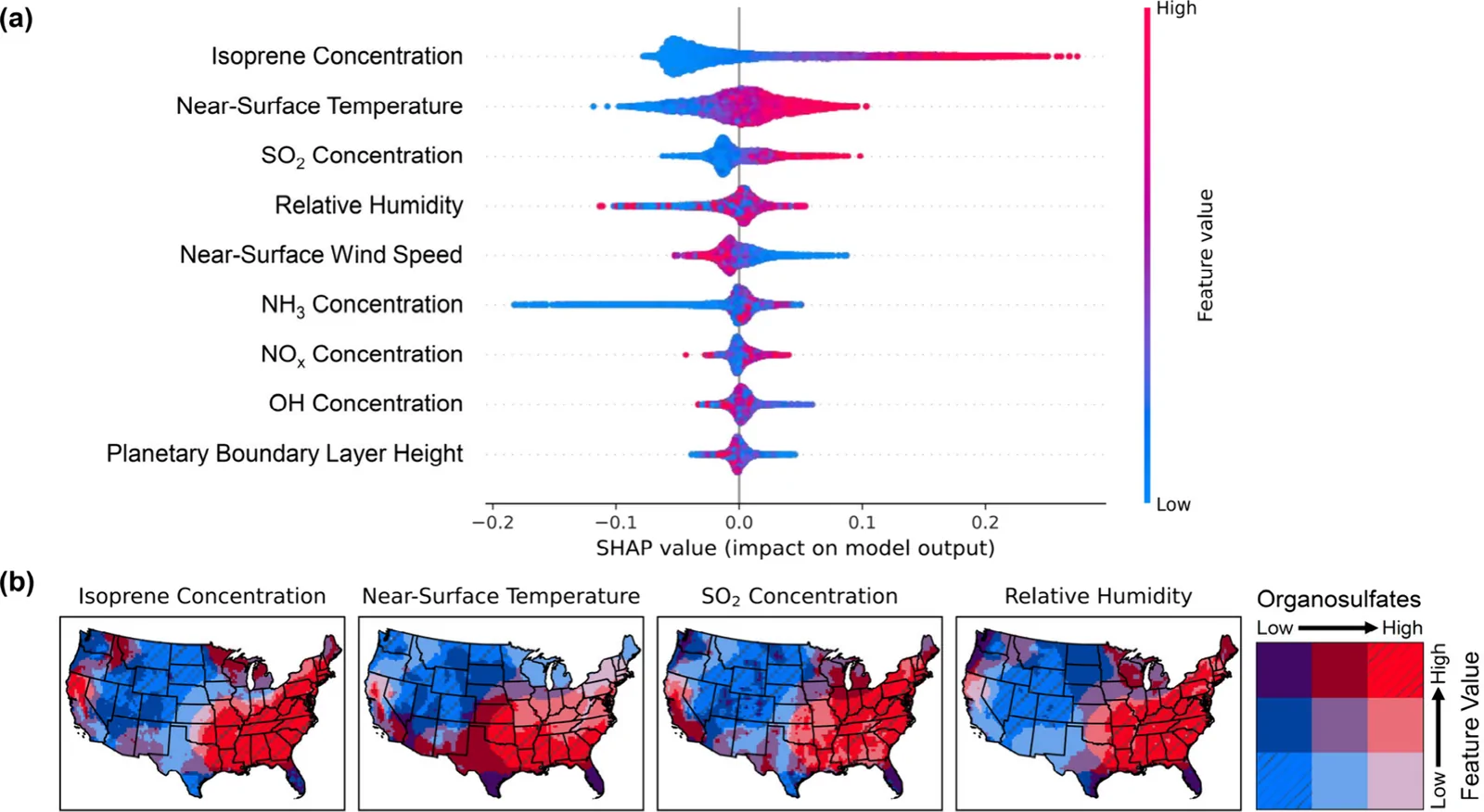
Contribution of primary predictive features to the modeled summertime concentrations of organosulfates. (a) The SHAP beeswarm plot for the XGBoost model shows the impact of each feature on the organosulfate concentrations, with features ranked by their mean absolute SHAP value (importance). Each point corresponds to a single prediction instance, with point color representing the original feature value and SHAP values on the *x*-axis indicating the contribution of a feature to the prediction (positive values increase the prediction, negative values decrease it). (b) Bivariate maps for the top performing features demonstrate the intersection between features and modeled organosulfate concentrations, with hatched areas indicating regions that exhibit strong interactions.

Bivariate maps (Figures 5b and S31) further illustrated the interactions between primary features and modeled OS concentrations, and summertime maps for 2013, 2016, 2019, and 2022 demonstrated the spatiotemporal trends of these predictive features (Figure S32). Spatially, the higher values of isoprene concentration, temperature, SO_2_ concentration, and relative humidity are associated with higher modeled OS concentration in the Southeast region; vice versa for the Mountains and Plains region. In contrast, higher OS concentration in northern California was primarily associated with higher isoprene concentrations. Temporally, isoprene concentrations, temperature, and relative humidity increased from June to July and August (Figures S33–S35), corresponding to generally higher OS concentrations in July/August. SO_2_ concentrations decreased across years in the Mid-Atlantic and Midwest regions (Figure S36), consistent with a decreasing interannual trend of OS concentrations.

### 3.5 Limitations and Future Work

In this study, a flexible and generalizable modeling framework was developed to quantify OSs across a large domain. The framework successfully captured the major spatial gradients and temporal variability in the observational constraints, supporting its use for a nationwide OS assessment. However, there are some limitations that are inherent to OS speciation, the availability of measurement data, and model inputs.

Isoprene-derived OSs (iOSs) were used as the modeled basis for estimating total OrgS and OS mass because they are among the best-characterized OS species, have the most available speciated measurement constraints, and can be estimated from CMAQ simulations with updated isoprene chemistry. However, iOSs represent only one component of total OrgS, and many other OrgS species and formation pathways remain poorly characterized and cannot yet be readily quantified at regional or national scales. To maximize consistency and coverage, we prioritized using IMPROVE-inferred OrgS as the primary nationwide observational constraint, rather than relying only on sparse field campaigns for speciated OS. OrgS was derived indirectly from measurements as the difference between total particulate sulfur and inorganic sulfate, and analogous OrgS estimates were derived from the model by converting modeled and adjusted iOSs to sulfur-equivalent mass and scaling them to total OrgS using literature-based, location-dependent iOrgS/OrgS fractions. This indirect, two-step comparison enables a nationwide evaluation with consistent long-term coverage, but it assumes that unquantified OrgS compounds are predominantly OSs (rather than other OrgS species like sulfonates, sulfides, and thiols) and that the speciation patterns represented by the iOrgS/OrgS fractions are broadly transferable across years and regions.

For direct evaluation, our modeled iOS concentrations agreed well with total iOS concentrations chemically characterized from IMPROVE filter samples, providing an important independent check on the iOS estimates.^35^ Consistent with other field campaigns,^23–25^ our results also showed that the low-NO_*x*_ pathway (IEPOX-OS) yielded more than 90% of IEPOX-OS + MPAN-OS concentrations. However, the CMAQ iOS parametrization used here was developed and constrained largely using laboratory and intensive field-campaign data from a limited number of sites, including 2013 SOAS. As a result, the raw CMAQ-simulated IEPOX-OS + MPAN-OS concentrations were closer to field-campaign-scale concentrations, which were substantially higher than those measured from IMPROVE filters. IEPOX-OS + MPAN-OS concentrations from the chemically characterized IMPROVE filter samples were about 20% of corresponding concentrations measured during SOAS and yielded an approximately 22% lower relative contribution to total iOSs.^23,35^ This contrast highlights an important scale and representativeness issue: field campaigns often capture more localized or episodic high-concentration conditions, whereas IMPROVE is designed primarily to characterize regional/background conditions over broader spatial and temporal scales. Recent studies evaluating OS stability, which is relevant to IMPROVE filter storage and analysis, have also shown that methyltetrol sulfate mass concentrations can decrease by 25% within the first 23 days, likely due to conversion into other iOSs, thereby potentially corresponding to the lower IEPOX-OS concentrations and relative contributions quantified from IMPROVE filter samples.^58^ Together, these factors suggest that peak iOS concentrations may be underestimated in locations less well represented by IMPROVE, and that IMPROVE-based iOS concentrations may also be affected by sample storage and stability.

To align the national, long-term analysis with IMPROVE constraints, we applied a postprocessing uniform adjustment factor of 0.2 to raw CMAQ-simulated iOS concentrations while preserving modeled spatial and temporal variability. Sensitivity tests showed that modeled OrgS magnitudes depend on the adjustment-factor assumption. The location-based sensitivity cases provided a useful test of spatially varying corrections, but their lower agreement with IMPROVE-derived OrgS suggests that directly correcting raw CMAQ-predicted iOS fields with IMPROVE-based measurements may introduce additional uncertainty and overcorrect spatial patterns, particularly because the CMAQ iOS parametrization was developed from laboratory and field-campaign constraints at a limited set of observational conditions. Additional field measurements near IMPROVE sites, particularly in areas such as northern California, where our results exhibit larger deviations, would help identify the causes of the differences between field-campaign, IMPROVE, and model-derived iOS concentrations. Such measurements would also support improved iOS adjustment approaches, including more robust location-based factors, and further constrain CMAQ iOS parameters, such as heterogeneous reaction rates and uptake coefficients. Consistent sampling methods and comprehensive OS quantification across regions will be important for this future evaluation.

Moreover, uncertainties in model inputs and configuration can propagate into OS formation, κ estimates, and CCN calculations. Both biogenic and anthropogenic emissions carry uncertainty in magnitude, speciation, and temporal allocation, which affects OS precursors and sulfate availability; we partially addressed this by conducting an NEI sensitivity simulation (in addition to EDGAR-based simulations) but remaining emissions uncertainty is unavoidable. In addition, the 36 km grid resolution may smooth subgrid gradients (especially in urban corridors and coastal regions), which can affect peak concentrations and localized hotspots. Our results targeted on summer months, when isoprene emissions are generally elevated and OS formation pathways are expected to be most active.^59^ While this seasonal focus does not capture year-round behavior, it likely represents a high-OS period and therefore provides an upper-bound estimate of OS burdens and their contributions to total κ. Estimation of κ relies on standard mixing-state and component-property assumptions, which involves uncertainties; however, our sensitivity tests indicate that uncertainty in κ_OS_ had a minor effect on total κ.

### 3.6 Implications

Although the focus of this study was to quantify and assess OSs in the U.S., readily available global data sets (e.g., ERA5 and EDGAR) were intentionally implemented to facilitate replicability of similar outputs and broader applications in other regions. In a wider context, our results highlight the importance of explicitly representing OS formation for accurately characterizing aerosol composition and climate-relevant properties, including aerosol hygroscopicity and CCN concentrations. As reported by Working Group I in the Intergovernmental Panel on Climate Change (IPCC) Sixth Assessment Report (AR6), aerosols remain the largest source of uncertainty in assessments of Earth’s radiative forcing.^60^ Because OS formation is not always included in CTM configurations, OS mass is often not simulated, and inorganic sulfate can be overestimated, particularly in regions such as the Southeast where emissions and meteorological conditions favor OS production. Consistent with this, our comparisons with IMPROVE observations showed that including OS formation reduced the overestimation of inorganic sulfate mass concentrations. In addition, OSs have a higher individual κ value than what is typically assigned to lumped organics. Together, missing OS and biased sulfate can lead to the overestimation of aerosol hygroscopicity and associated climate-relevant effects. Specifically, an overestimated total κ can overpredict aerosol water uptake and wet-particle growth, leading to higher simulated scattering/extinction and AOD.^9,10^ It can also overestimate CCN concentrations, as supported by our supplemental CCN calculations, and thereby potentially overestimate aerosol cooling from aerosol–radiation and aerosol–cloud interactions.^9–11^ Including OS formation therefore improves the physical consistency of aerosol composition and κ estimates, which helps reduce uncertainty in AOD and CCN-related climate effects.

The machine-learning attribution and SHAP analysis further indicate that OS variability is statistically associated with both meteorological factors (e.g., temperature and relative humidity) and precursor concentrations (e.g., isoprene and SO_2_). Although SHAP does not establish causality, the identified predictive features are chemically plausible and consistent with established OS formation pathways. For example, higher isoprene concentration increases precursor availability for iOS, and higher temperature enhances biogenic isoprene emissions and photochemical production of reactive isoprene oxidation products. Meanwhile, higher SO_2_ increases sulfate abundance and aerosol acidity, while higher relative humidity increases aerosol liquid water, creating favorable conditions for reactive uptake and multiphase OS formation.^61^ These mechanisms are consistent with the enhanced OS concentrations in the Southeast U.S., where warm, humid conditions coincide with abundant isoprene and sulfate-related aerosol chemistry. Prior Southeast U.S. studies have shown that declining anthropogenic SO_2_ emissions reshaped the regional aerosol system by reducing sulfate, aerosol liquid water, and associated aerosol optical/radiative effects.^62–64^ This broader regional context helps explain, in part, the decreasing OS concentrations from 2013 to 2022 in this study. More broadly, the statistical associations and mechanistic context indicate that future changes in OSs and κ should be interpreted in the context of concurrent changes in temperature-dependent biogenic VOC emissions and photochemistry, anthropogenic sulfur emissions, humidity, aerosol liquid water content, and acidity.^34,65,66^ Although some predictors, such as NO_*x*_ and NH_3_, exhibit relatively lower marginal importance in our trained models, emission changes for these species may still affect climate through other pathways.^67,68^

This study has several strengths with scientific and policy relevance: (1) by integrating updated isoprene-derived OS chemistry in CMAQ with an IMPROVE-based evaluation framework, it provides the first comprehensive, observation-validated data set of OS concentrations with high spatial and temporal (hourly; multiple years) coverage across the U.S.; (2) by pairing simulations with and without OS formation, it delivers the first nationwide quantification of both the direct and indirect impacts of OSs on κ; (3) it couples these mechanistic results with an interpretable machine-learning surrogate that both identifies dominant predictive features and enables rapid estimation of OS concentrations without rerunning CMAQ. Together, these capabilities support timely screening of how emission controls of key precursors and climate variability may alter OS concentrations, sulfate partitioning, and CCN-relevant hygroscopicity, supporting both management of particulate matter and assessment of aerosol radiative and cloud-mediated climate effects.

## Supplementary Material



## Notes

Model domain and regions; emissions processing; modeled organosulfates; evaluation of modeled organosulfur and inorganic sulfate with IMPROVE measurements; sensitivity tests; fractional contributions and relative changes in aerosol hygroscopicity (κ); CCN calculations; and attribution through machine learning (PDF)
