# Supplementary material for: Modeling Decadal Organosulfates in Particulate Matter to Improve Estimations of Aerosol Hygroscopicity in the United States
Source: Environ Sci Technol. 2026 Nov 2;60(44):31786–97. doi: 10.1021/acs.est.6c08752 (PMC13757952; doi:10.1021/acs.est.6c08752)
Supplement: Supplementary file 1 [file es6c08752_si_001.pdf]

Supporting Information

Modeling Decadal Organosulfates in Particulate Matter to Improve Estimations of Aerosol Hygroscopicity in the United States

Alan R. Gonzalez[1], Heewon Yim[1], Kyle P. McCary[2], Xiangxinyue Meng[2], Ajith Thenoor Chandran[2], Yue Zhang[2], Yuzhou Wang[1,*]

[1] Zachry Department of Civil and Environmental Engineering, Texas A&M University, College Station, Texas 77843, United States

[2] Department of Atmospheric Sciences, Texas A&M University, College Station, Texas 77843, United States

[*] Corresponding author: Yuzhou Wang (yuzhouw@tamu.edu)

Content includes:

Text S1 to S3

Tables S1 and S2

Figures S1 to S36

References

Text S1. CMAQ Modeling of Isoprene-Derived Organosulfates

For our CMAQ simulations, we used the SAPRC-11 gas-phase chemical mechanism with additional isoprene oxidation chemistry as described by Xie et al.[1] and Lin et al.[2] and modeled isoprene-derived SOA through reactive surface uptake processes. In the CMAQ modeling scheme and as shown in Figure 1b, isoprene undergoes oxidation through reactions with hydroxyl radicals (OH) and hydroperoxyl radicals (HO_2) to produce isoprene epoxydiols (IEPOX) under low-NO_x conditions.[3,4] Under high-NO_x conditions, the oxidation of isoprene yields methacryloylperoxynitrate (MPAN) and subsequently produces methacrylic acid epoxide (MAE) and hydroxymethyl-methyl-α-lactone (HMML).[5,6] The heterogeneous uptake of gas-phase isoprene epoxides (IEPOX and MAE + HMML) onto aqueous aerosols is parameterized through an uptake coefficient (γ) based on a pseudo-first-order particle-phase rate constant, Henry's Law coefficient, and additional molecular properties that yield isoprene SOA.[7–11] The resulting aerosol-phase organosulfate products from the low- and high-NOx pathways are represented in this study as IEPOX-OS and MPAN-OS, respectively. Thus, our CMAQ simulations provided raw iOS (IEPOX-OS + MPAN-OS) estimates that responded dynamically to changing atmospheric conditions (i.e., through coupled chemistry and transport) while accounting for gas-particle partitioning and reactive loss processes.

Text S2. iOS Adjustment Factors and Sensitivity Tests

The base-case analysis applied a uniform adjustment factor of 0.2 to raw CMAQ-predicted IEPOX-OS + MPAN-OS concentrations, as described in the main text. This factor was used to adjust the overall magnitude of CMAQ-predicted iOS concentrations to the scale of the chemically characterized 2016 IMPROVE filter measurements while retaining the spatial and temporal variability simulated by CMAQ.

To evaluate sensitivity to this adjustment-factor assumption, we considered four additional cases (Table S1). Two cases used uniform lower- and upper-bound adjustment factors of 0.16 and 0.35. The lower-bound case considered the average IEPOX-OS + MPAN-OS concentration at the three IMPROVE sites nearest to Centreville, AL, which are expected to be lower than concentrations in Centreville, AL. The upper-bound case considered the maximum total iOS concentration observed across all IMPROVE sites to represent how IEPOX-OS + MPAN-OS concentrations constitute a significant fraction of total iOS concentrations but correspond to different relative contributions when comparing measurements from the 2016 IMPROVE sites and 2013 SOAS.

The other two cases used location-based adjustment factors. For each chemically characterized 2016 IMPROVE site, we calculated a site-specific adjustment factor as the ratio between the IMPROVE-based iOS measurement and the corresponding raw CMAQ-predicted IEPOX-OS + MPAN-OS concentration. The lower-bound location-based case used measured IEPOX-OS + MPAN-OS as the IMPROVE-based constraint, yielding adjustment factors ranging from 0.00 to 0.24, with an average of 0.07 (Figure S3a). The upper-bound location-based case used total measured iOS concentrations across all quantified species, yielding adjustment factors ranging from 0.02 to 0.36, with an average of 0.15 (Figure S4a). The site-specific factors were then interpolated across the modeling domain using linear radial basis function interpolation.

Text S3. CCN Calculation and Response to OS-Induced Hygroscopicity Changes

To further assess how OS-induced changes in aerosol hygroscopicity affect cloud condensation nuclei (CCN), we calculated fine-mode CCN concentrations using modeled aerosol number-size distributions and κ values for the baseline case without OS formation (κ_{base}) and the OS-inclusive case ($\kappa_{\mathrm{w/OS}}$). Calculations were performed at critical supersaturations (s_c) of 0.1%, 0.4%, and 0.8%, which represent the minimum water vapor supersaturation required for particles to activate into cloud droplets.

Based on κ–Köhler theory[12] and as shown in eqs S1 and S2, aerosol particles activate into cloud droplets when their dry diameter exceeds a critical diameter (D_{crit}):

$$D_{crit} = \left(\frac{4A^3}{27\kappa \ln^2 S_c}\right)^{1/3} \quad \text{(S1)}$$

$$A = \frac{4\sigma_{s/a} M_w}{RT\rho_w} \quad \text{(S2)}$$

where S_c is the critical saturation ratio corresponding to s_c (1.001, 1.004, and 1.008), A is the curvature (Kelvin) effect, $\sigma_{s/a}$ is droplet surface tension (0.0728 N m^{-1}), M_w is the molecular weight of water (18.01528 g mol^{-1}), R is the universal gas constant (8.3145 J mol^{-1} K^{-1}), T is the temperature obtained from the meteorological inputs, and ρ_w is the density of water (1000 kg m^{-3}).

The CCN number concentration for each CMAQ fine aerosol mode (CCN_i) was determined by integrating the corresponding particle number-size distribution above the critical diameter, and the resulting CCN concentrations were summed across both modes to obtain the total fine-mode CCN concentration ($\mathrm{CCN}_{\mathrm{fine}}$) using eqs S3 and S4:

$$\mathrm{CCN}_i = \int_{D_{crit}}^{\infty} n_i(D)dD \quad \text{(S3)}$$

$$\mathrm{CCN}_{\mathrm{fine}} = \mathrm{CCN}_{\mathrm{AIT}} + \mathrm{CCN}_{\mathrm{ACC}} \quad \text{(S4)}$$

where $n_i(D)$ is the lognormal particle number size distribution for either the Aitken (AIT) or accumulation (ACC) mode.

Across the selected supersaturations, fine-mode CCN concentrations were highest in the eastern U.S. and including OSs in κ calculations resulted in a consistent CCN decrease (Figure S26). The response was strongest at s_c = 0.1%, with an average decrease of 6.2% and a maximum decrease of 13.3% across the U.S. Average decreases were smaller at s_c = 0.4% and 0.8%, at 2.6% and 1.6%, respectively. The stronger response at lower supersaturation reflects the greater sensitivity of CCN activation to aerosol hygroscopicity under these conditions. Under otherwise similar aerosol mass and size distributions, a decrease in κ can suppress hygroscopic growth and reduce aerosol scattering and extinction at a given relative humidity, while reduced CCN activation may weaken aerosol-cloud cooling. These effects would tend to reduce the predominantly cooling influence of aerosol-radiation and aerosol-cloud interactions.

Table S1. Sensitivity analyses of iOS adjustment factor effects on model performance relative to IMPROVE OrgS site-average concentrations (2013-2022) and changes in κ due to the inclusion of OSs.

Case	Description	Adjustment Factor	Model Performance		Changes in κ		
			Linear Regression Slope	R^2	Total Change (%)	Direct Change (%)	Indirect Change (%)
Base	Ratio of upper bound IEPOX-OS + MPAN-OS concentrations across IMPROVE sites (2016)[13] and average IEPOX-OS + MPAN-OS concentrations in Centreville, AL (2013)[14]	$\frac{140}{676} = 0.20$	0.802	0.823	-5.56	+2.82	-8.38
Lower- Bound	Ratio of IEPOX-OS + MPAN-OS average concentrations at the three nearest IMPROVE sites (GRSM1, MING1, and SHRO1; 2016)[13] and average IEPOX-OS + MPAN-OS concentrations in Centreville, AL (2013)[14]	$\frac{112}{676} = 0.16$	0.641	0.823	-4.52	+2.30	-6.82
Upper- Bound	Ratio of upper bound total iOS concentrations across IMPROVE sites (2016)[13] and average IEPOX-OS + MPAN-OS concentrations in Centreville, AL (2013)[14]	$\frac{236}{676} = 0.35$	1.403	0.823	-9.19	+4.55	-13.74
Lower-Bound, Location-Based	Ratio of IEPOX-OS + MPAN-OS concentrations at each IMPROVE site (2016)[13] and IEPOX-OS + MPAN-OS concentrations from the 2016 raw model output	0.07 (0.00–0.24)*, Figure S3	0.364	0.731	-2.42	+1.26	-3.68
Upper-Bound, Location-Based	Ratio of total iOS concentrations at each IMPROVE site (2016)[13] and IEPOX-OS + MPAN-OS concentrations from the 2016 raw model output	0.15 (0.02–0.36)*, Figure S4	0.678	0.777	-4.50	+2.31	-6.81

Note: IEPOX-OS and MPAN-OS correspond to measured concentrations (ng m^{-3}) of methyltetrol sulfate and 2-methylglyceric acid sulfate, respectively.
* Adjustment factors are presented as mean (range), with range indicating the minimum–maximum values.

Table S2. Modeled concentrations of iOSs for selected sites and dates in July/August 2016 and comparisons with measurements from IMPROVE filter samples.

Site	iOS Concentration (ng m^{-3})
ACAD1	78.09
BOND1	69.61
FRES1	60.66
GRSM1	128.35
LOND1	63.33
LYEB1	93.87
MING1	218.29
ORPI1	14.06
PORE1	18.82
PRIS1	64.74
PUSO1	25.25
REDW1	43.35
ROMO1	21.76
SHRO1	298.34
SNPA1	36.70
TALL1	25.93
ULBE1	8.54
WHPA1	4.01
**Model Average**	**71.39**
**Observed Average***	**69.24**
**Bias (%)**	**3.11**

* Calculated using national average concentrations of total iOSs from Chen et al. (Table S3)[13].

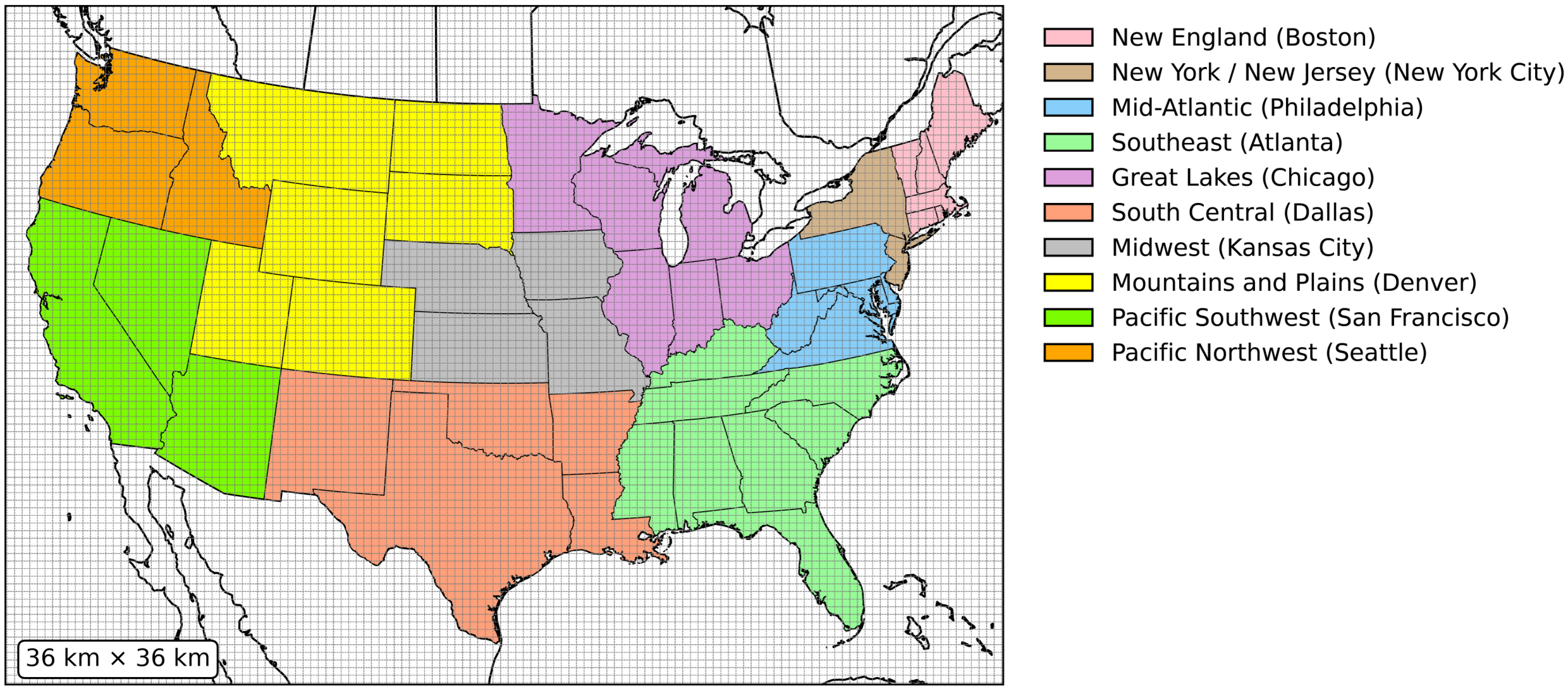

Figure S1. Map of model domain and regions.

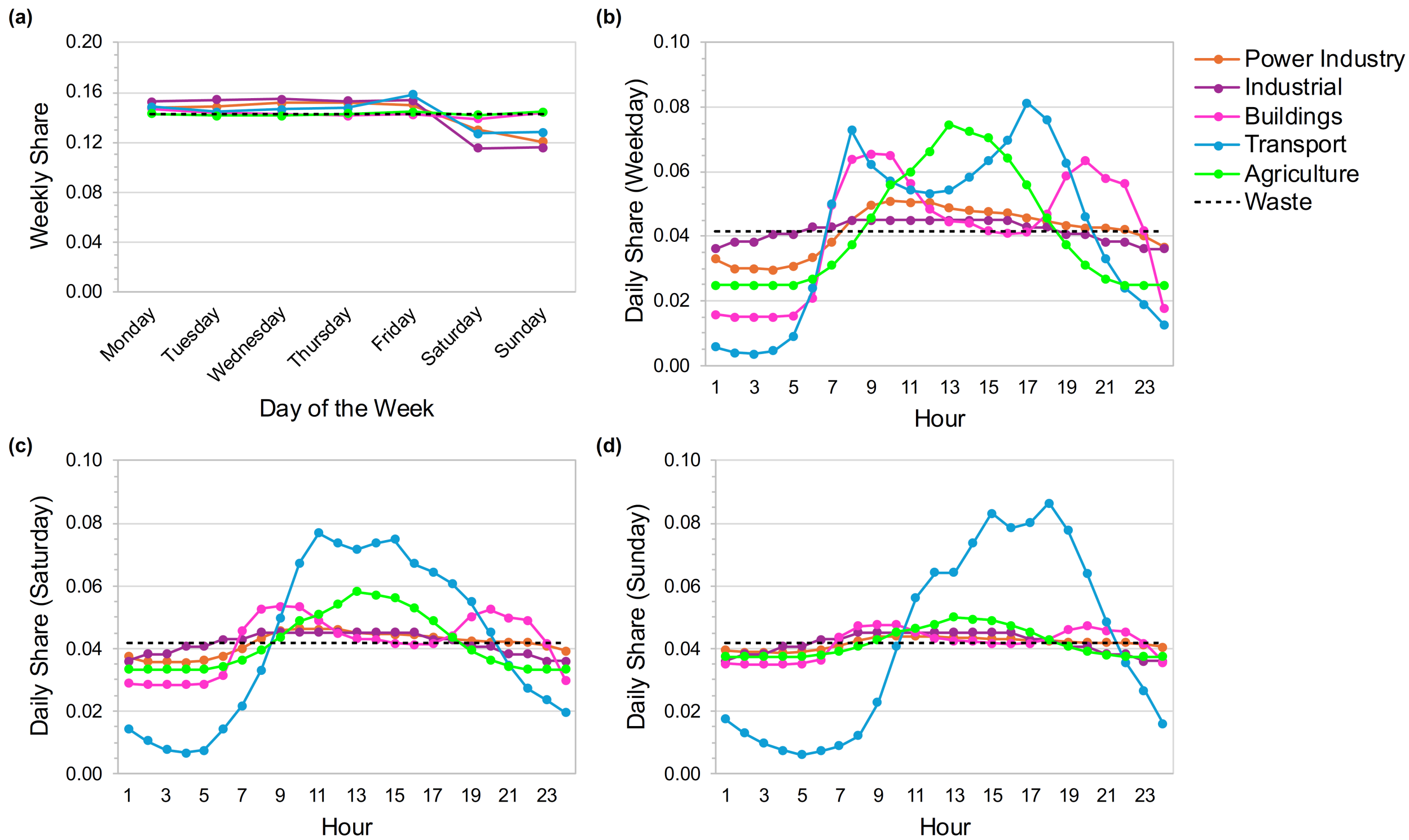

Figure S2. Sector-specific temporal profiles used to prepare hourly anthropogenic emissions. Monthly emissions were allocated to an hourly resolution based on (a) weekly and (b-d) diurnal profiles.

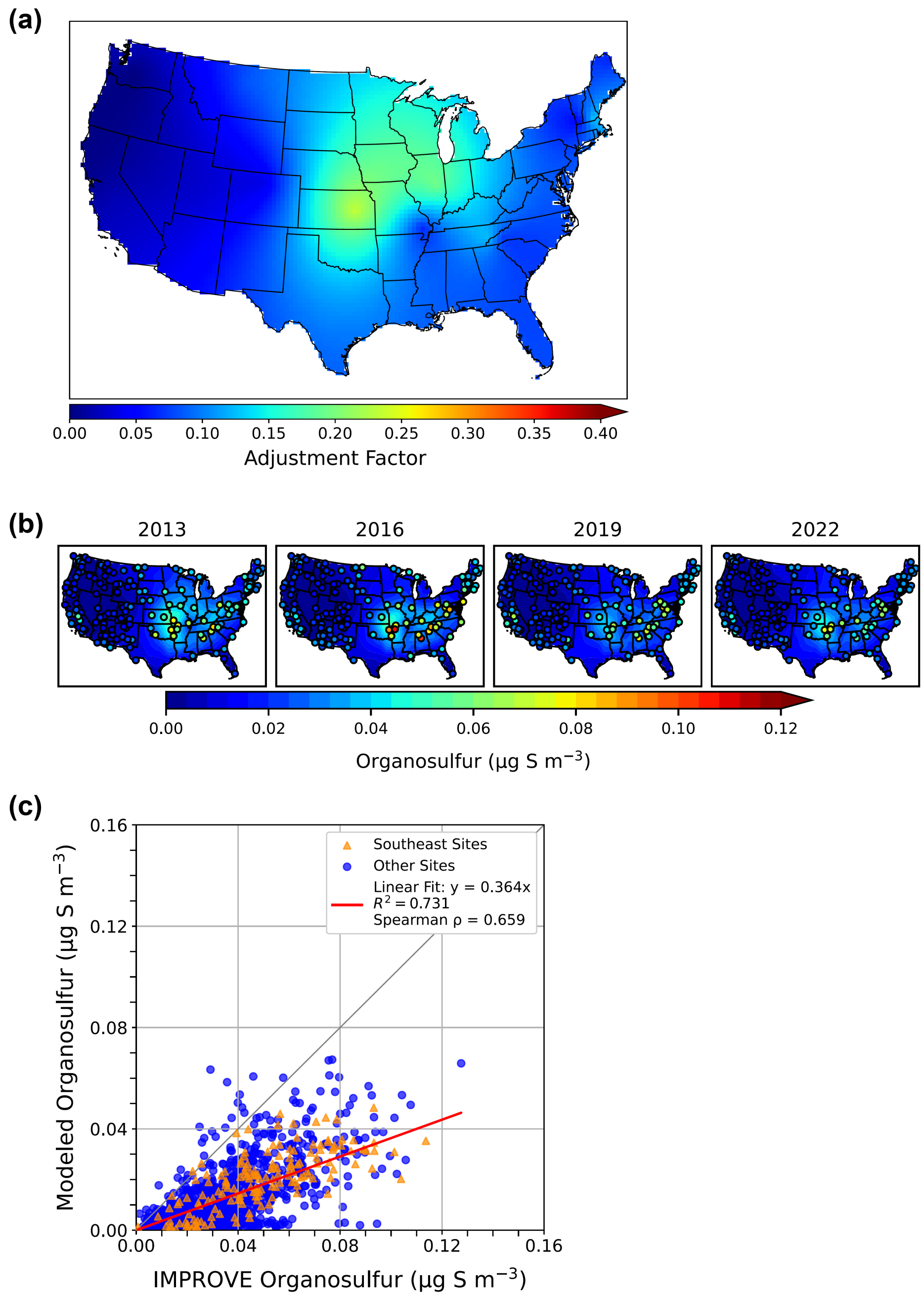

Figure S3. Sensitivity test that used the lower-bound, location-based adjustment factors to address differences between SOAS and IMPROVE IEPOX-OS + MPAN-OS concentrations. Plots include (a) the location-based adjustment factors used as input for the test, (b) evaluation of modeled OrgS concentrations with IMPROVE site data, and (c) a scatter plot of monthly average concentrations per site.

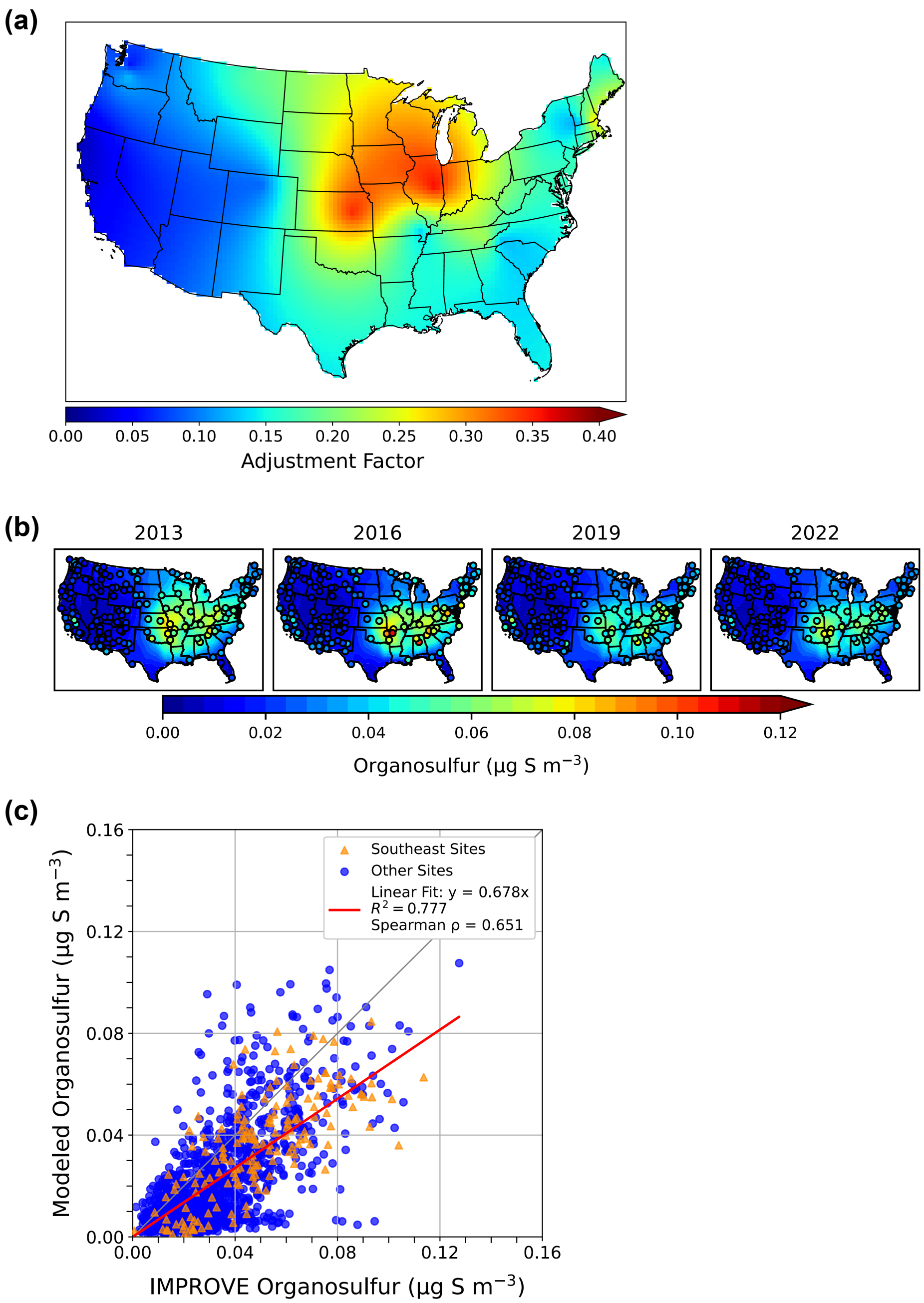

Figure S4. Analogous to Figure S3 but for the sensitivity test using the upper-bound, location-based adjustment factors.

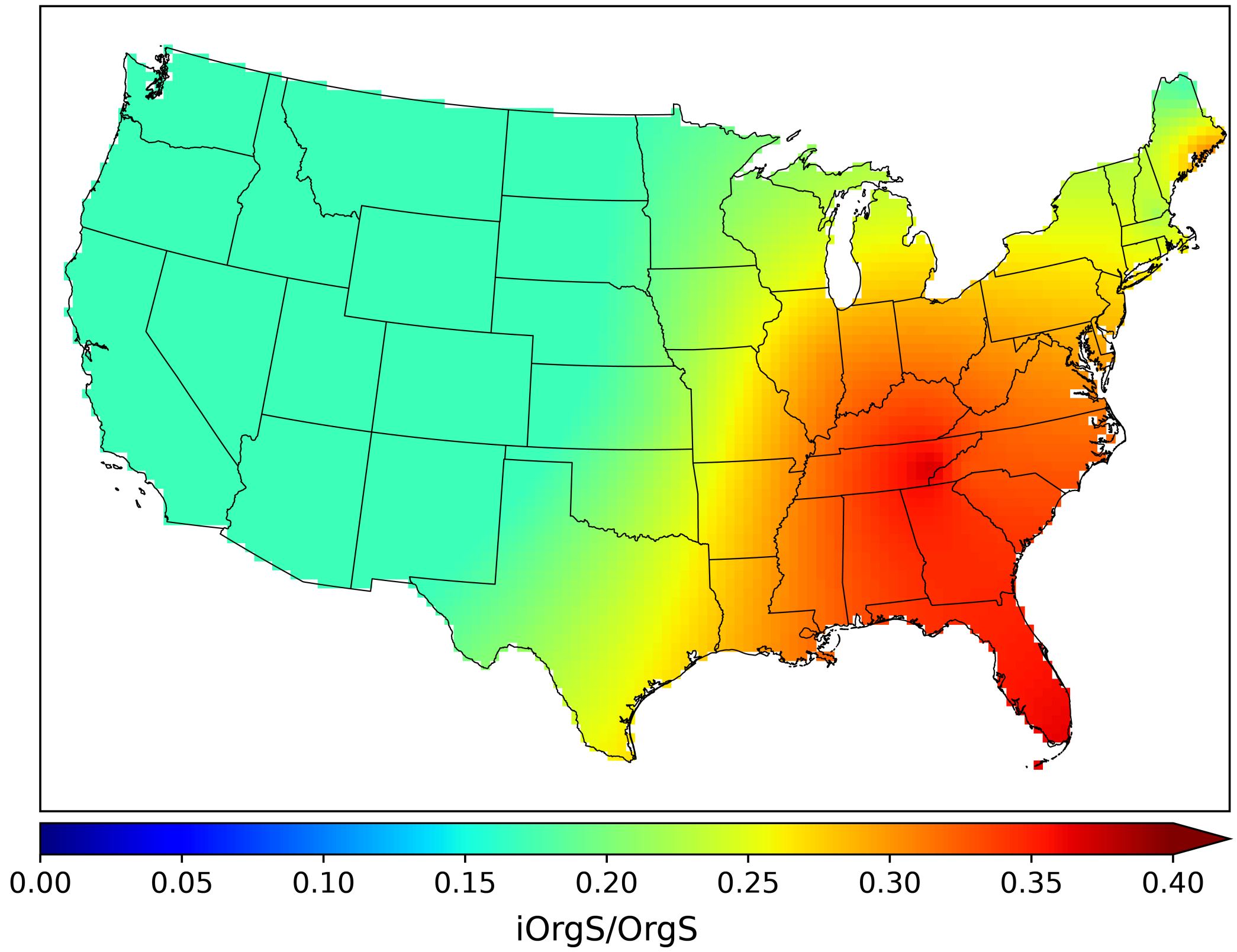

Figure S5. Location-dependent scaling factors used to calculate total OrgS from iOrgS (minimum threshold = 0.17; maximum = 0.37).

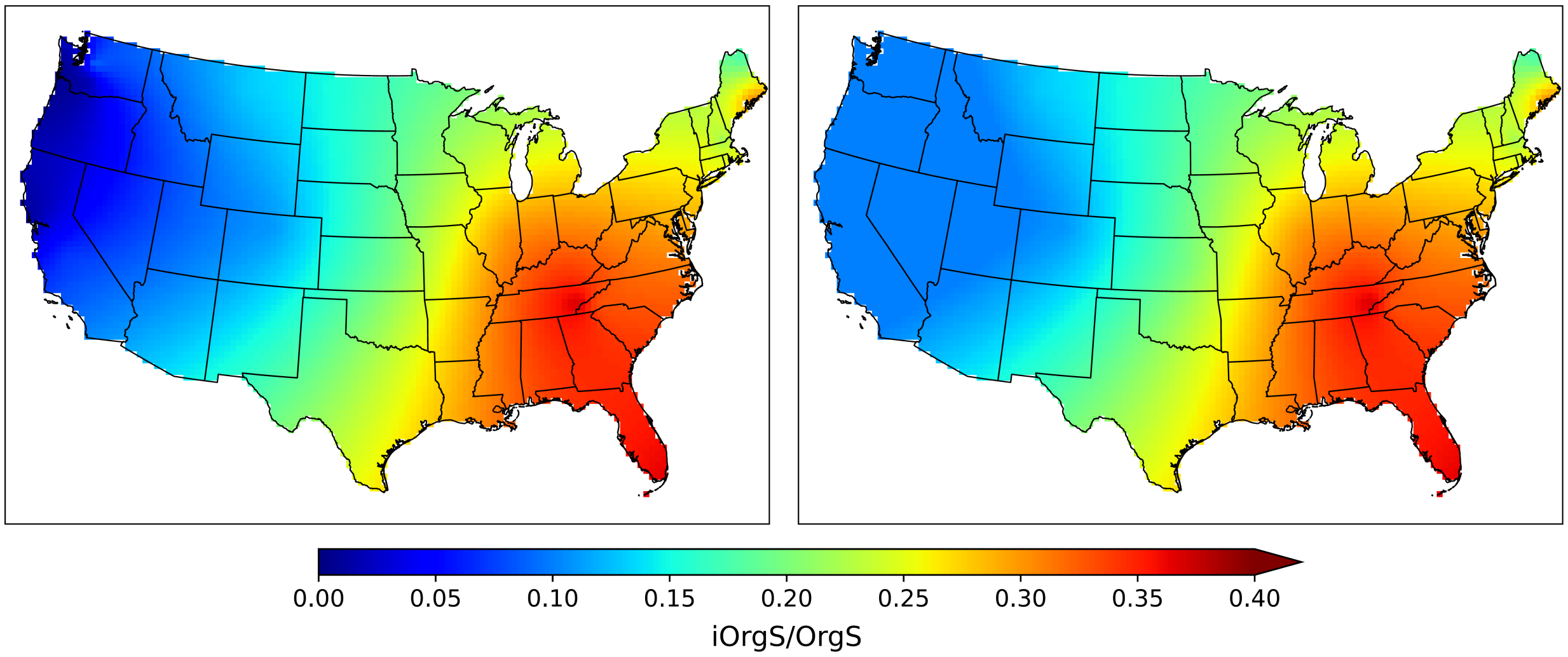

Figure S6. Location-dependent iOrgS/OrgS scaling factors for sensitivity tests. The figure on the left side shows the gridded iOrgS/OrgS field with no lower bound; the figure on the right side shows the gridded iOrgS/OrgS field with a lower bound of 0.1.

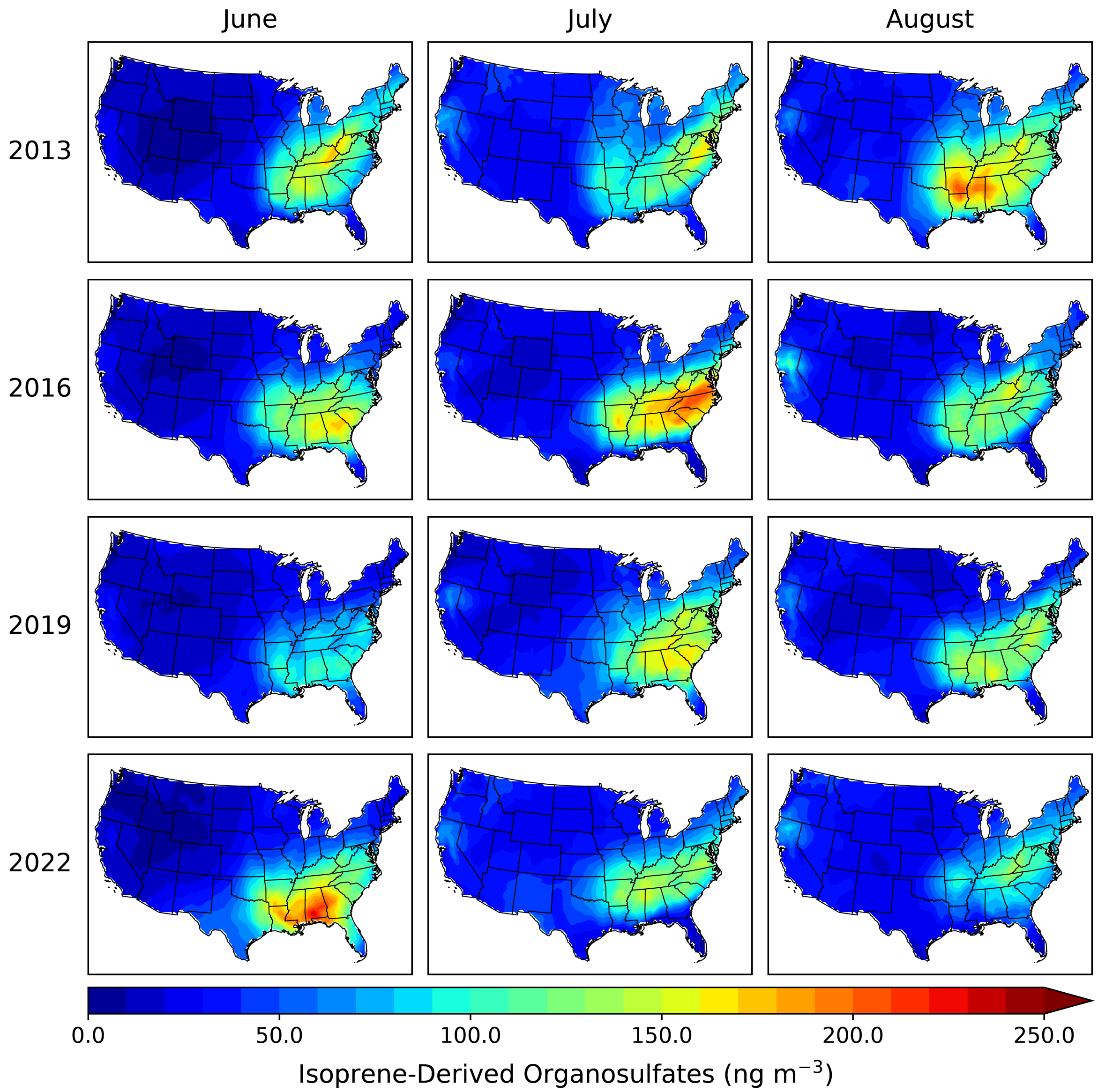

Figure S7. Monthly concentrations of iOS_{model}.

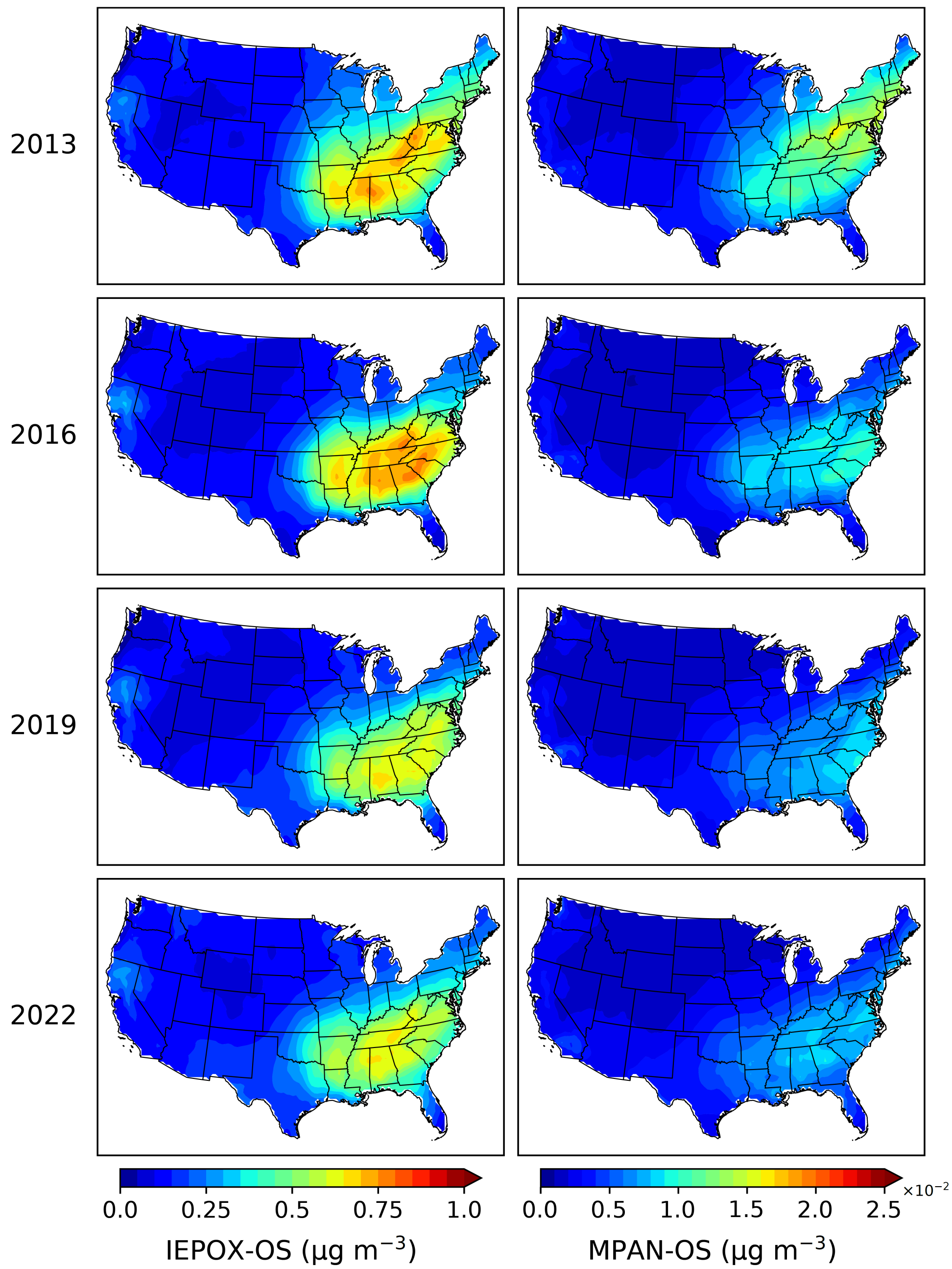

Figure S8. Yearly average particulate concentrations of IEPOX-OS and MPAN-OS.

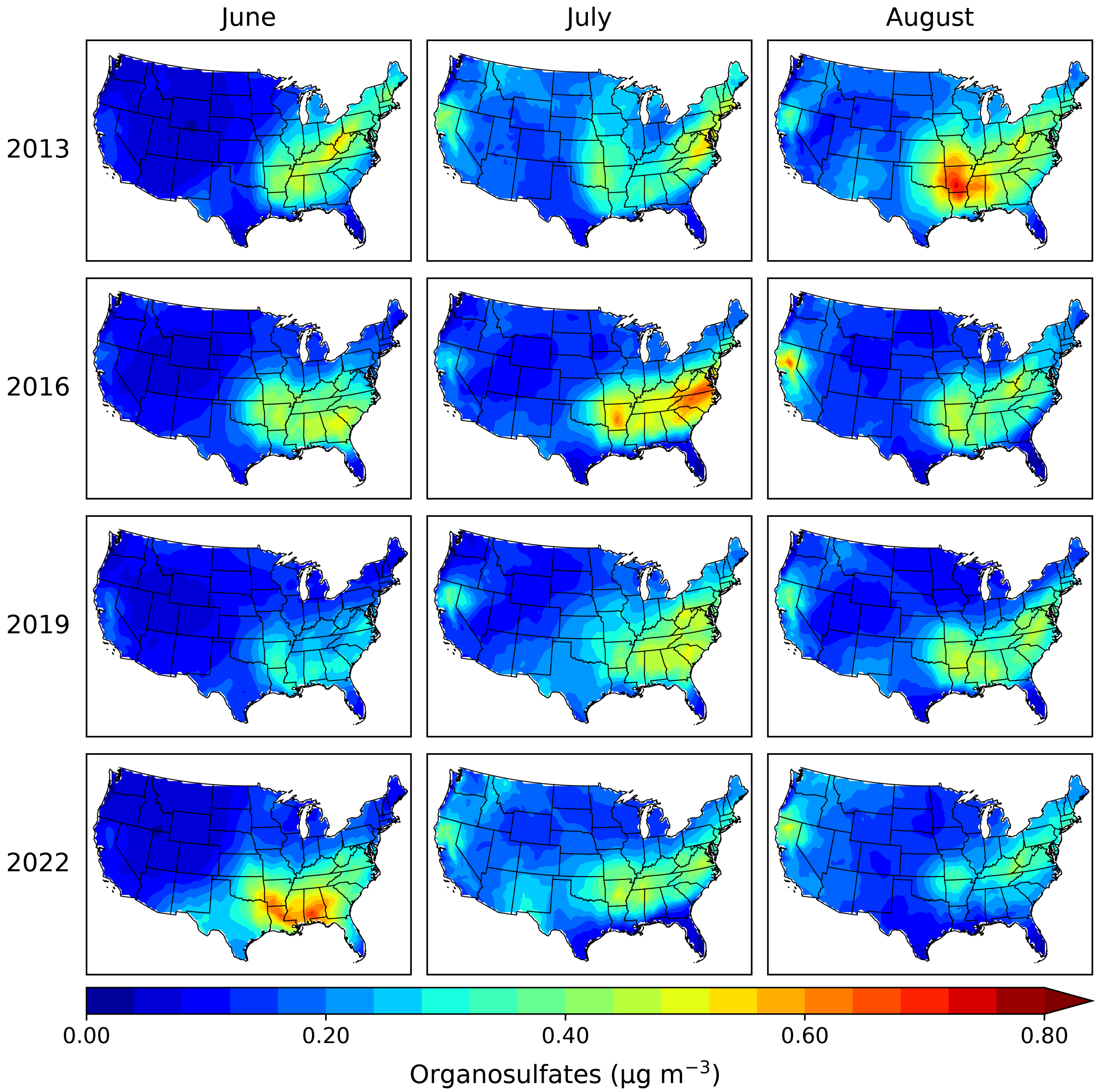

Figure S9. Monthly concentrations of OS_{model}.

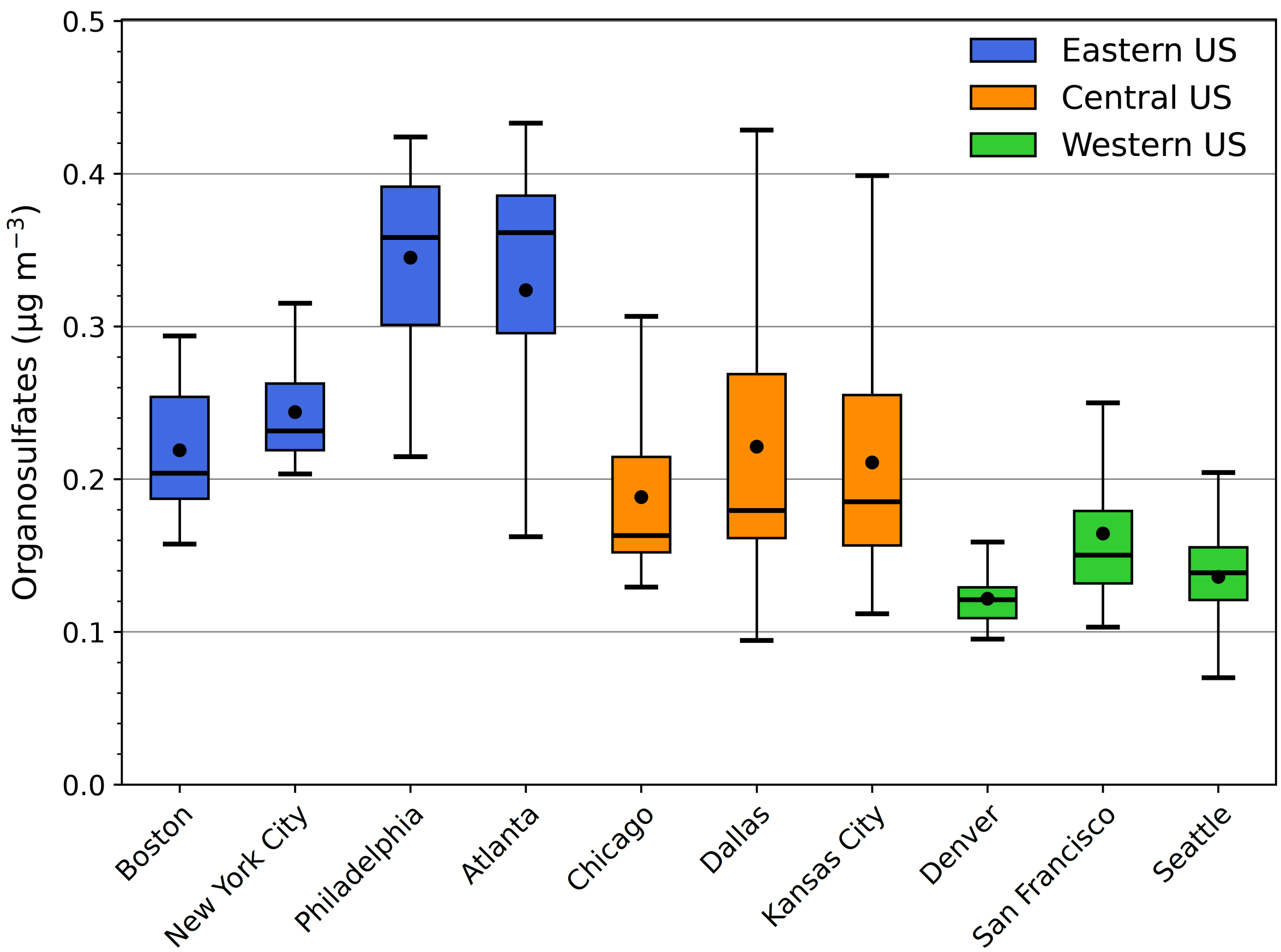

Figure S10. Average summertime concentrations of OSs across all modeled years (2013, 2016, 2019, and 2022) based on region. Boxes represent the interquartile range (IQR), extending from the first quartile (Q1) to the third quartile (Q3). Whiskers extend to the most extreme values within 1.5 times the IQR. The mean and median for each region are shown as a black dot and line, respectively.

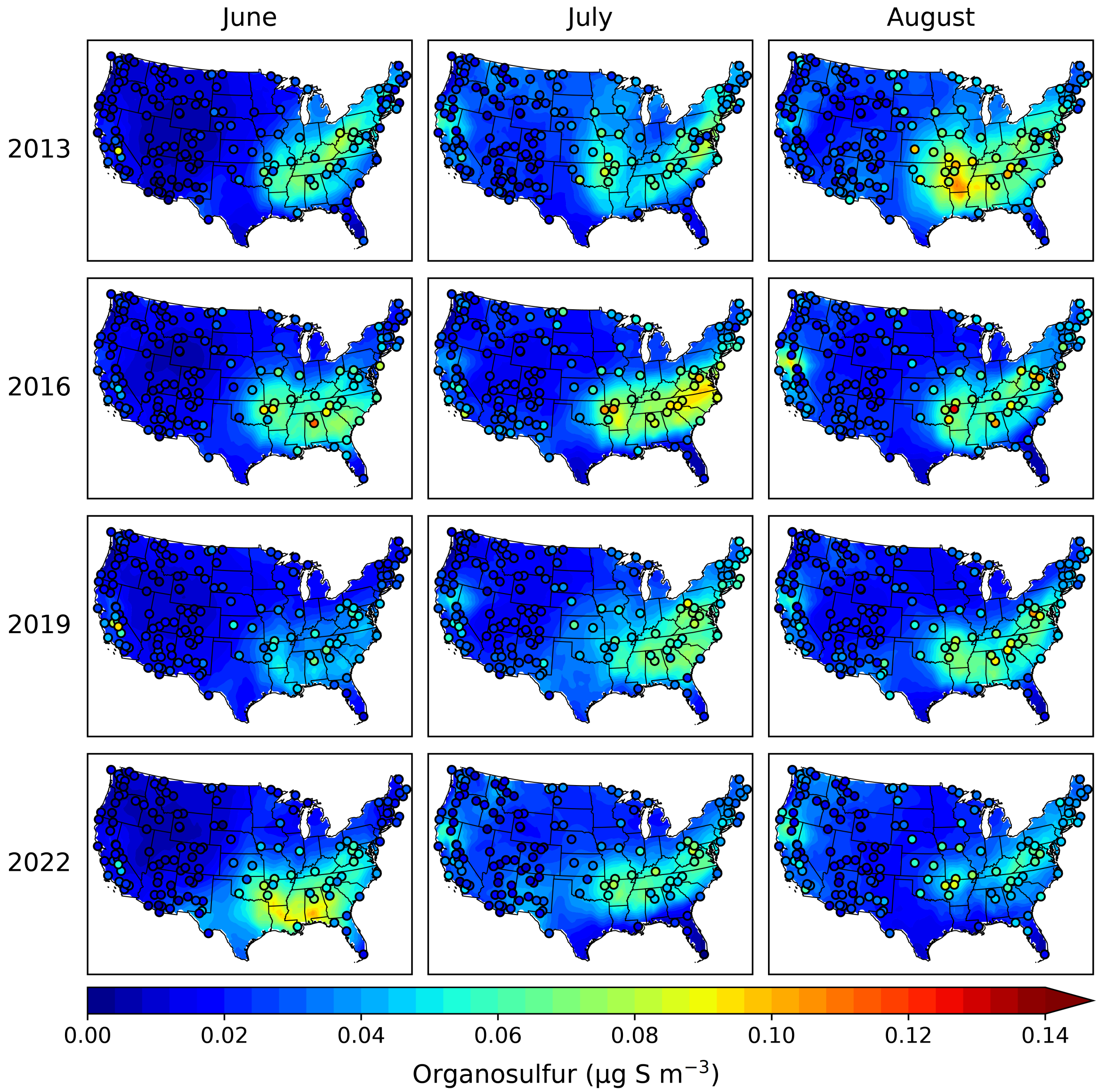

Figure S11. Monthly comparisons of $OrgS_{model}$ concentrations (background color) with measurement data from IMPROVE sites (points).

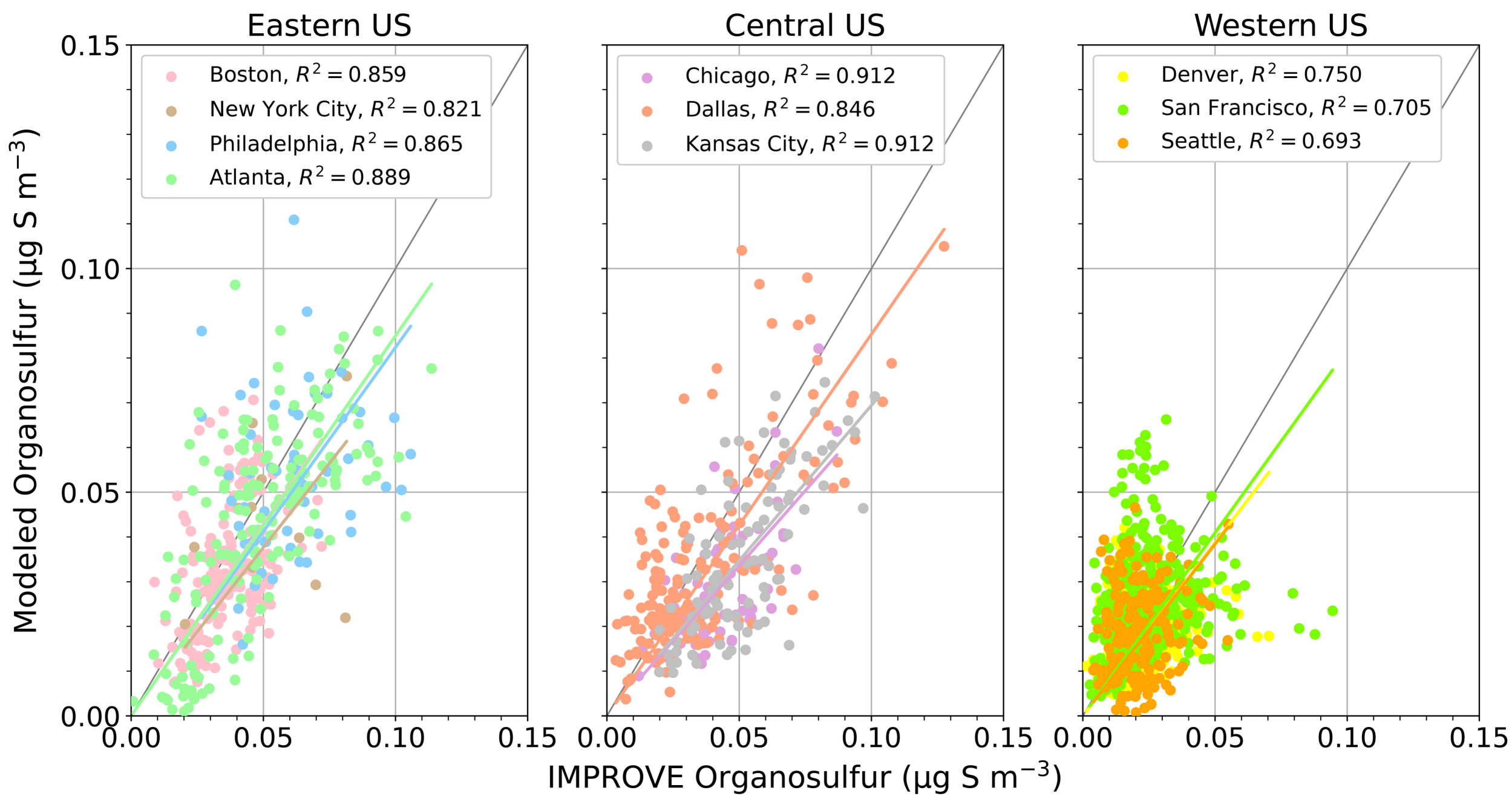

Figure S12. Scatter plot comparisons of modeled and IMPROVE OrgS concentrations for each U.S. region, each point representing the monthly average each year at a site.

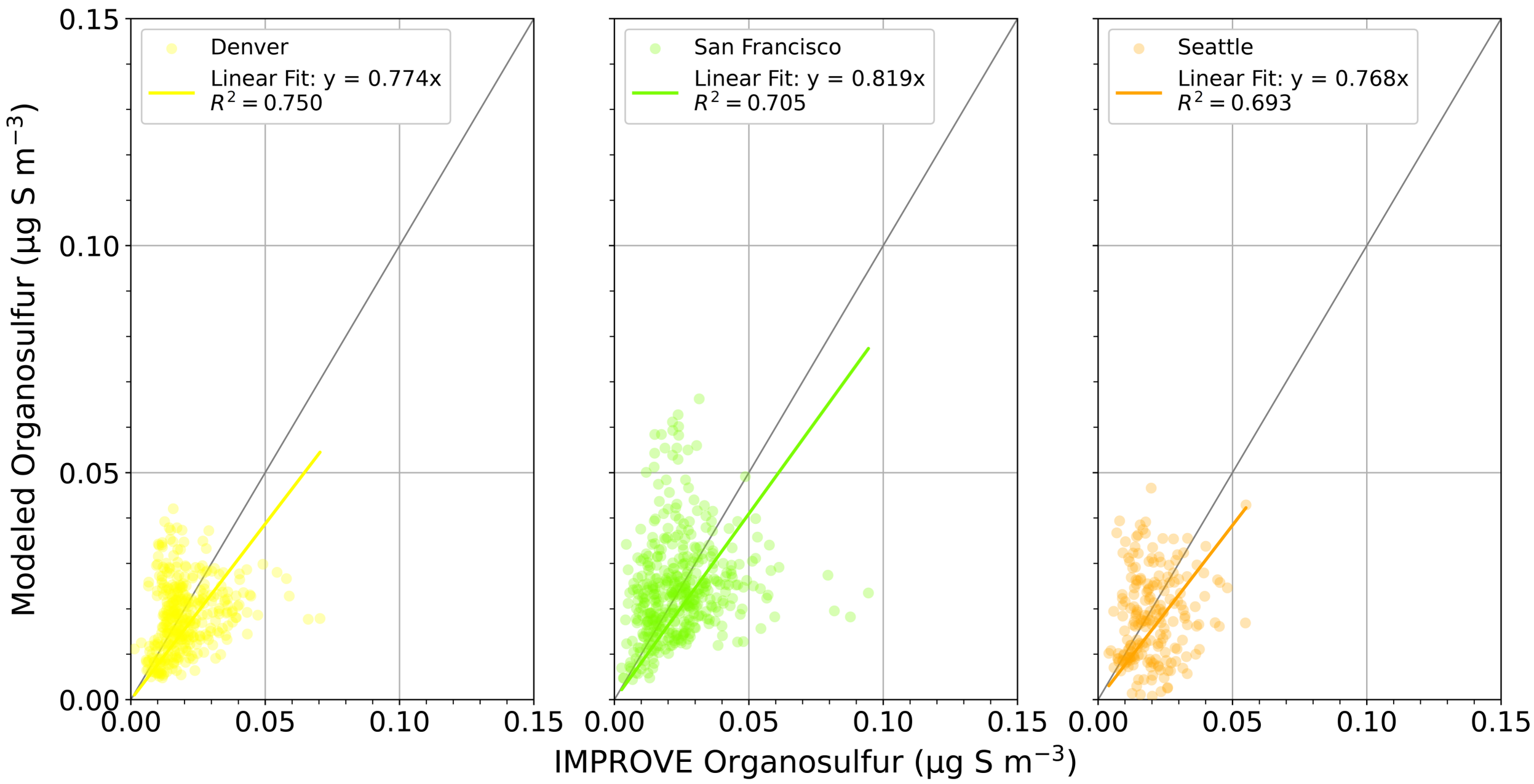

Figure S13. Scatter plot comparisons of modeled and IMPROVE OrgS concentrations for each EPA region in the western U.S., with each point representing the monthly average each year at a site.

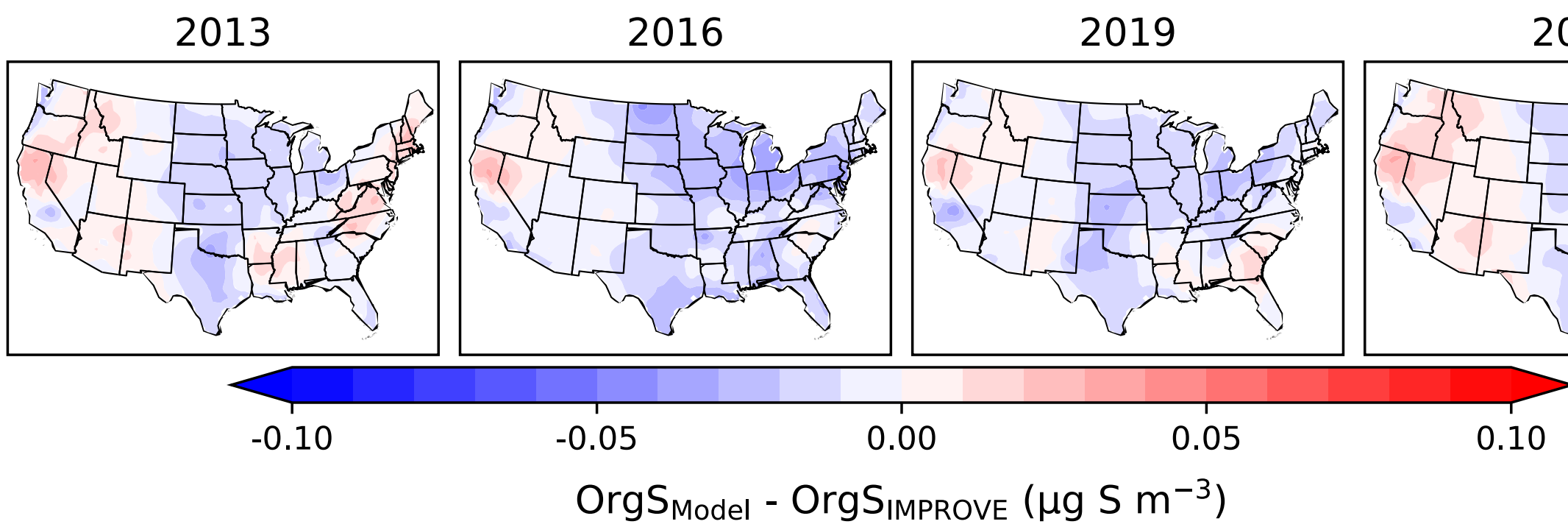

Figure S14. Yearly differences in modeled and IMPROVE OrgS concentrations.

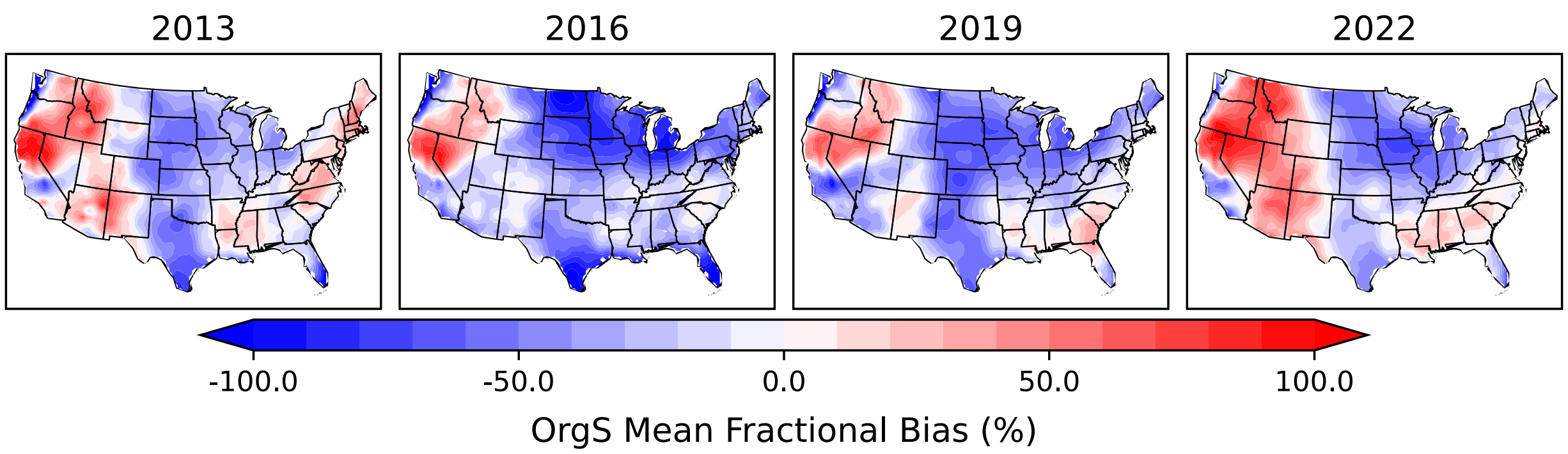

Figure S15. Mean fractional bias maps for modeled and IMPROVE OrgS concentrations.

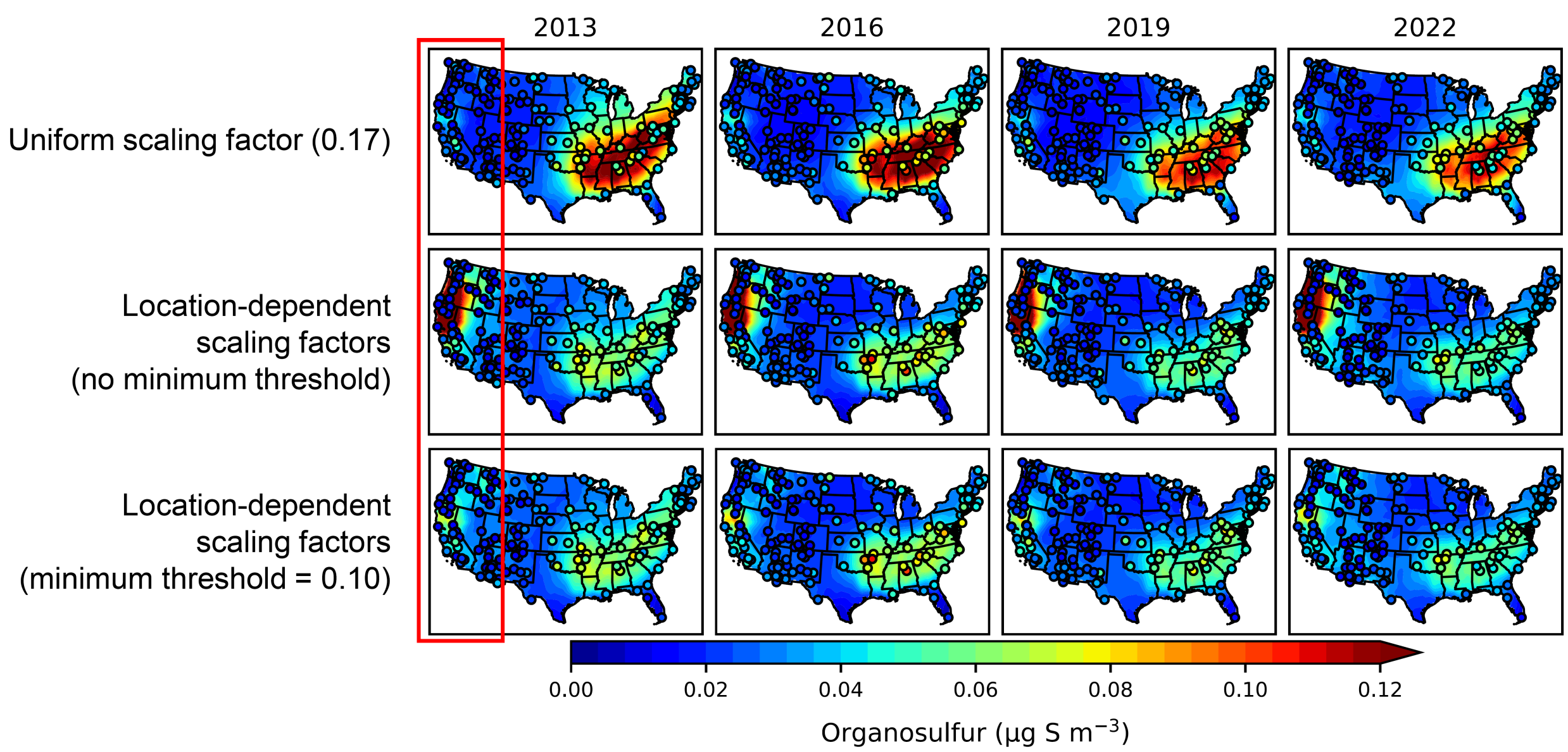

Figure S16. Additional sensitivity tests used to configure the iOrgS/OrgS scaling factors. Each map includes modeled OrgS concentrations (background color) and measurement data from IMPROVE sites (points). Red rectangle highlighted the Western U.S., where applying location-dependent scaling factors show more bias compared with measurements.

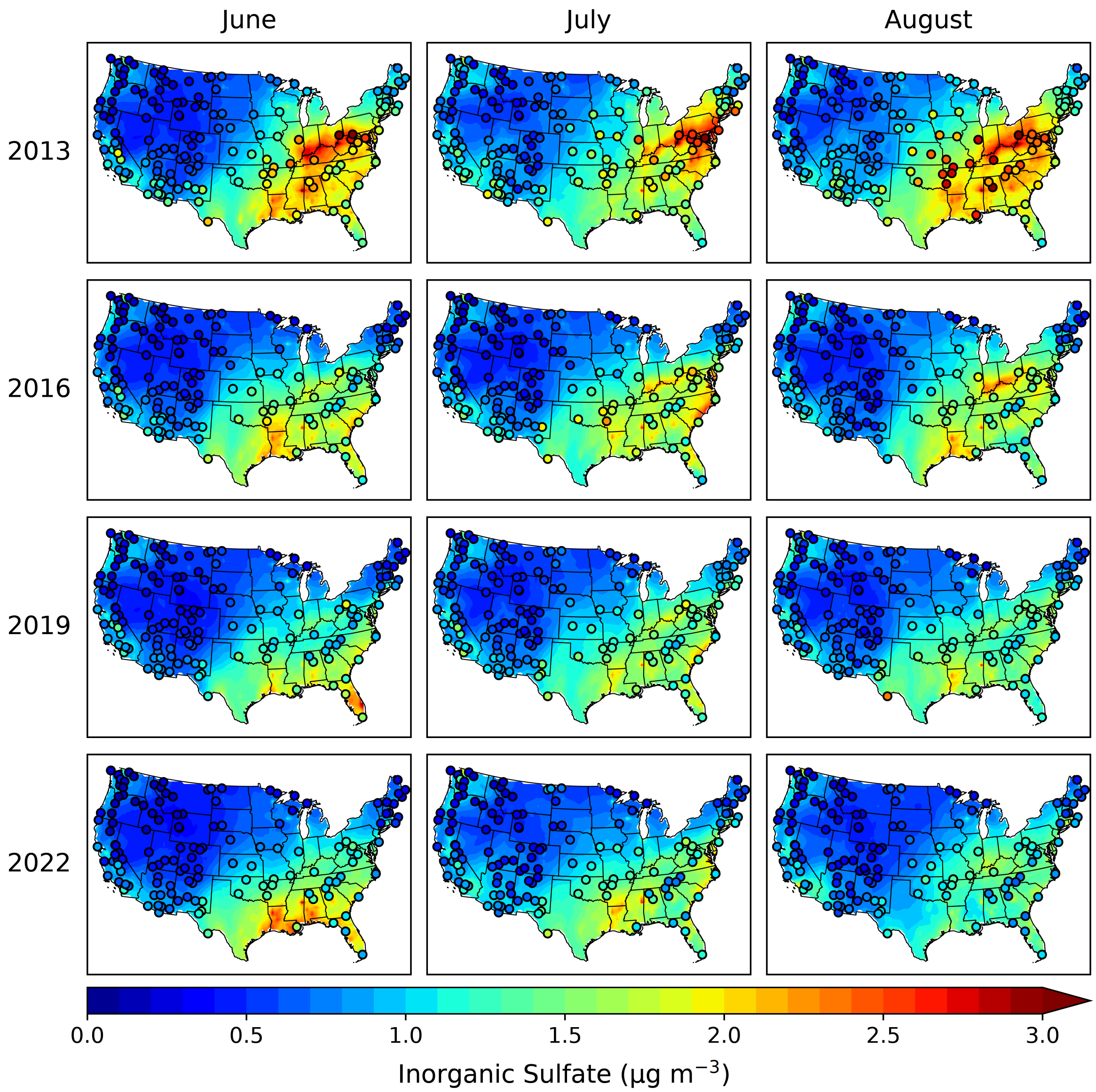

Figure S17. Monthly comparisons of modeled inorganic SO_4^{2-} concentrations (background color) with measurement data from IMPROVE sites (points).

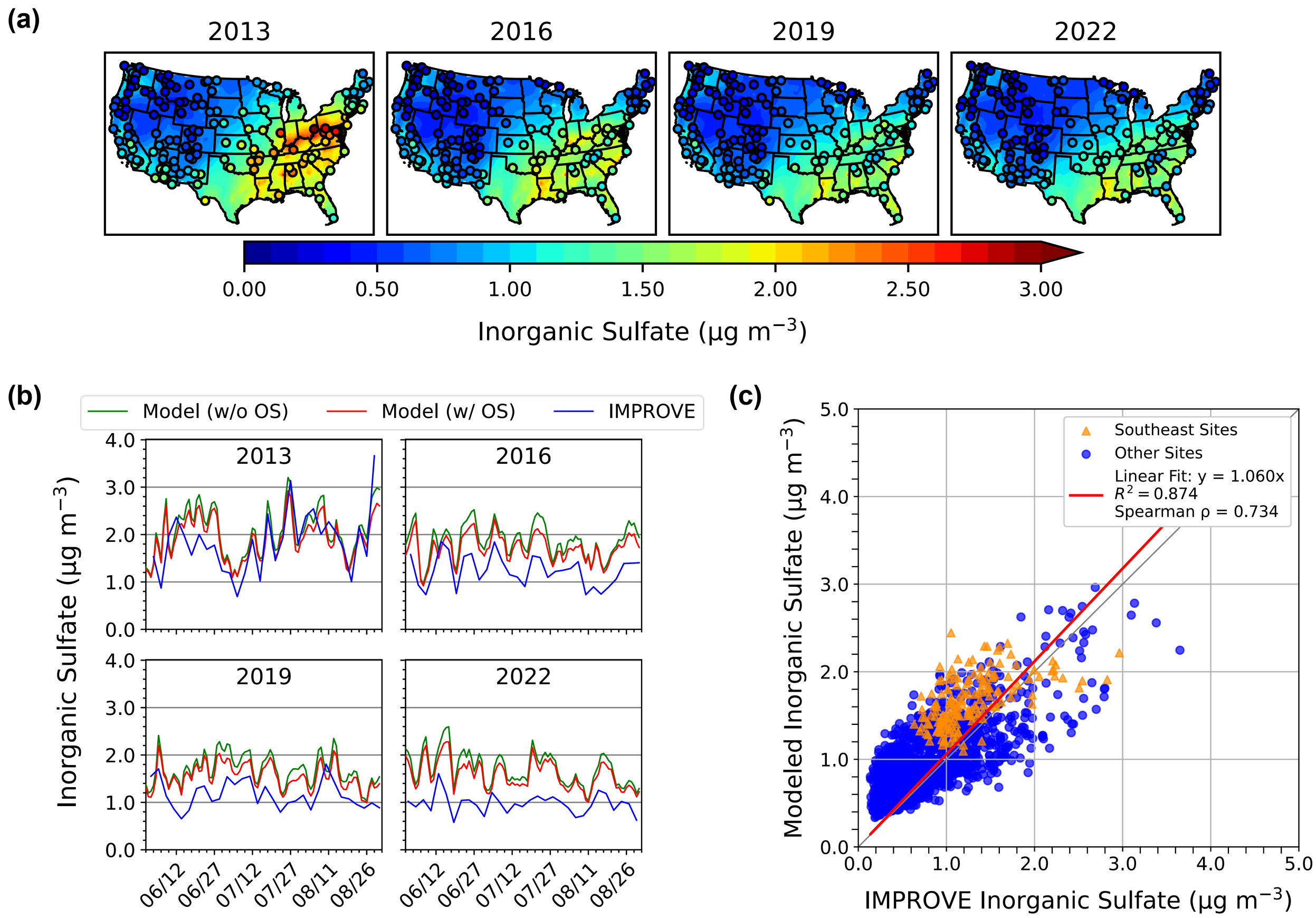

Figure S18. Evaluation of modeled inorganic SO_4^{2-} concentrations with IMPROVE site data. Evaluation includes (a) maps of average summertime concentrations, (b) time series comparisons of average concentrations in the Southeast region, and (c) a scatter plot of monthly average concentrations per site.

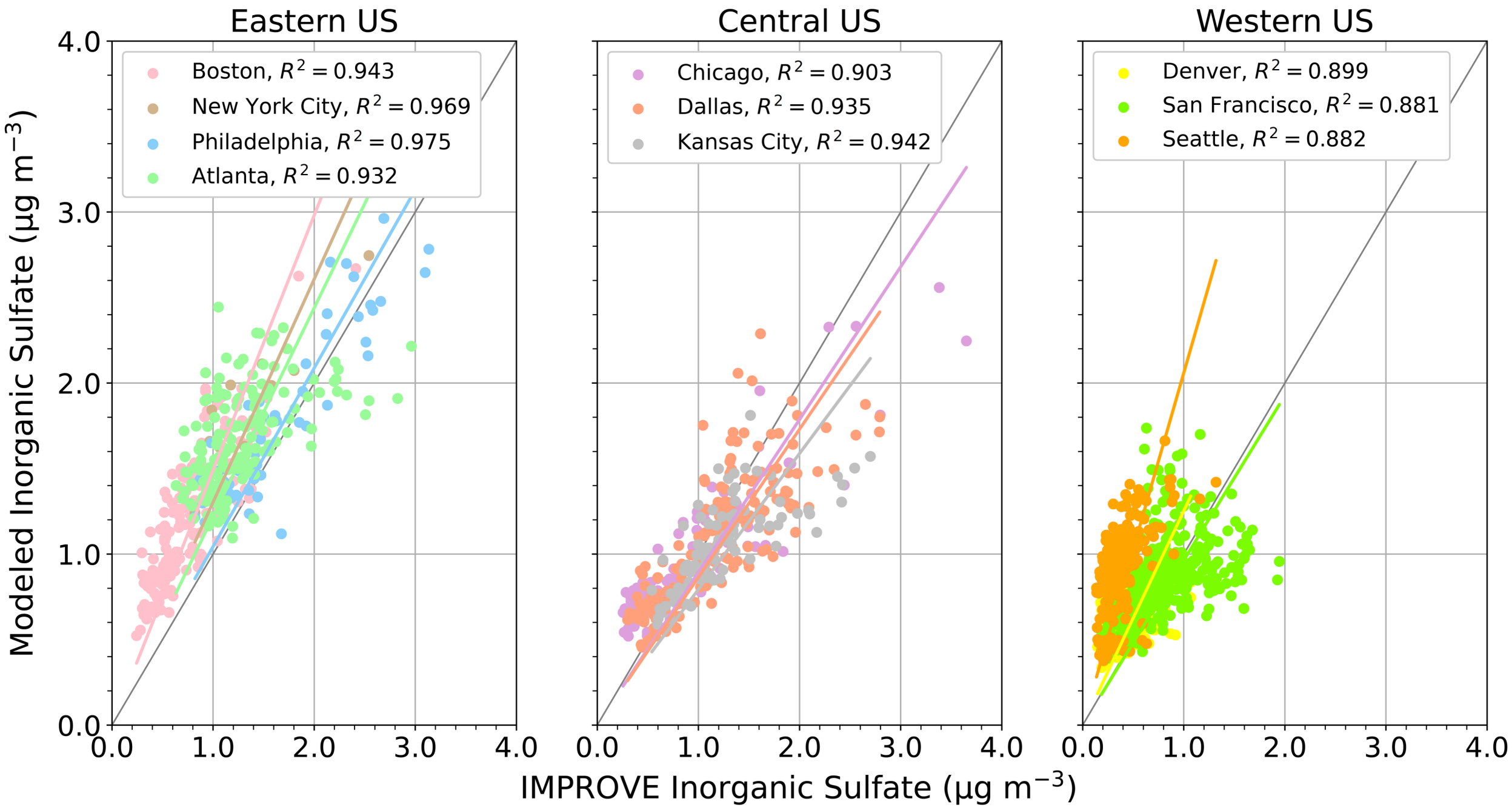

Figure S19. Scatter plot comparisons of modeled and IMPROVE inorganic SO_4^{2-} concentrations, each point representing the monthly average each year at a site.

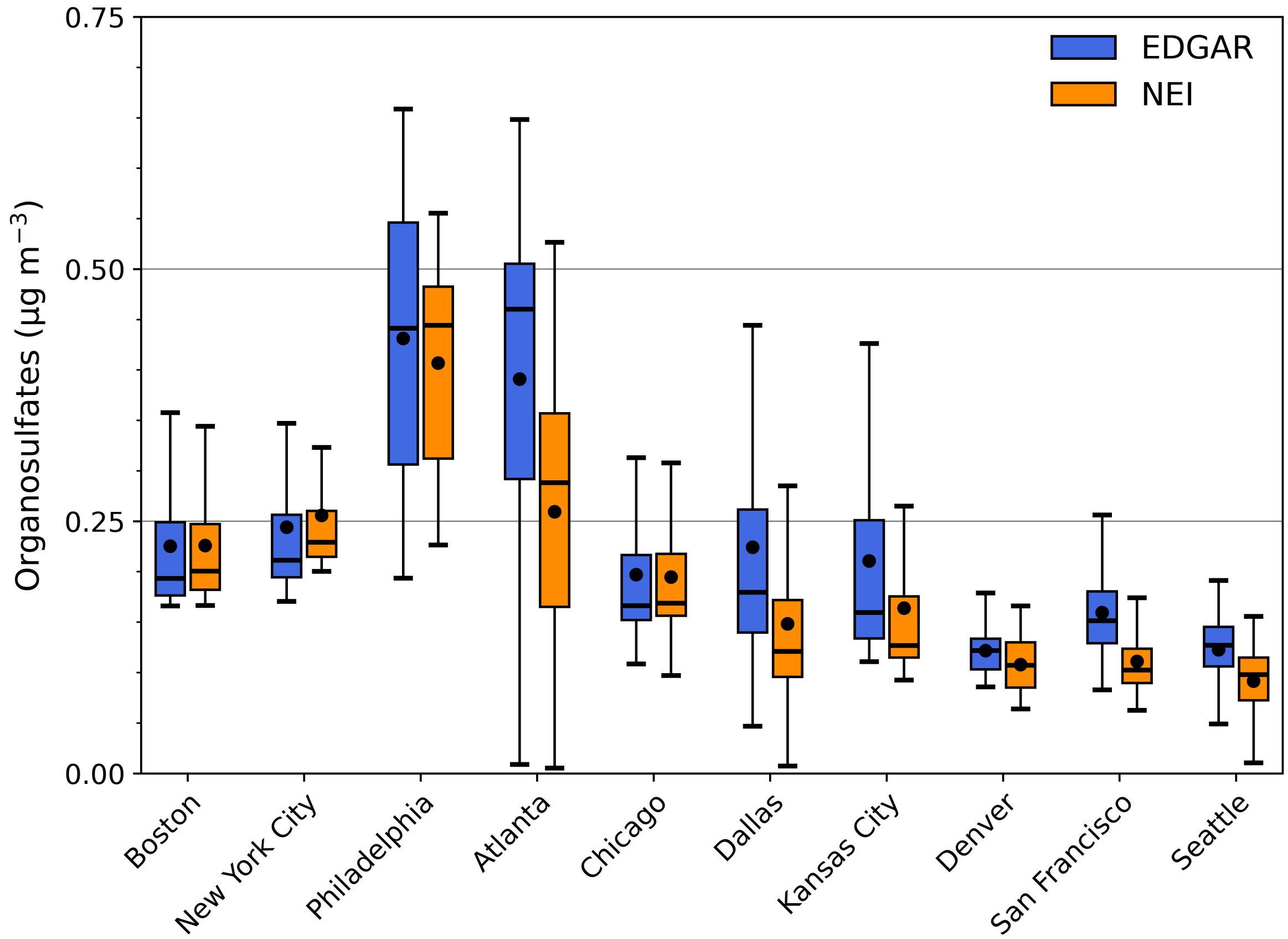

Figure S20. Average concentrations of OSs in July 2016 based on region and source of anthropogenic emissions (EDGAR and NEI). Boxes represent the interquartile range (IQR), extending from the first quartile (Q1) to the third quartile (Q3). Whiskers extend to the most extreme values within 1.5 times the IQR. The mean and median for each region are shown as a black dot and line, respectively.

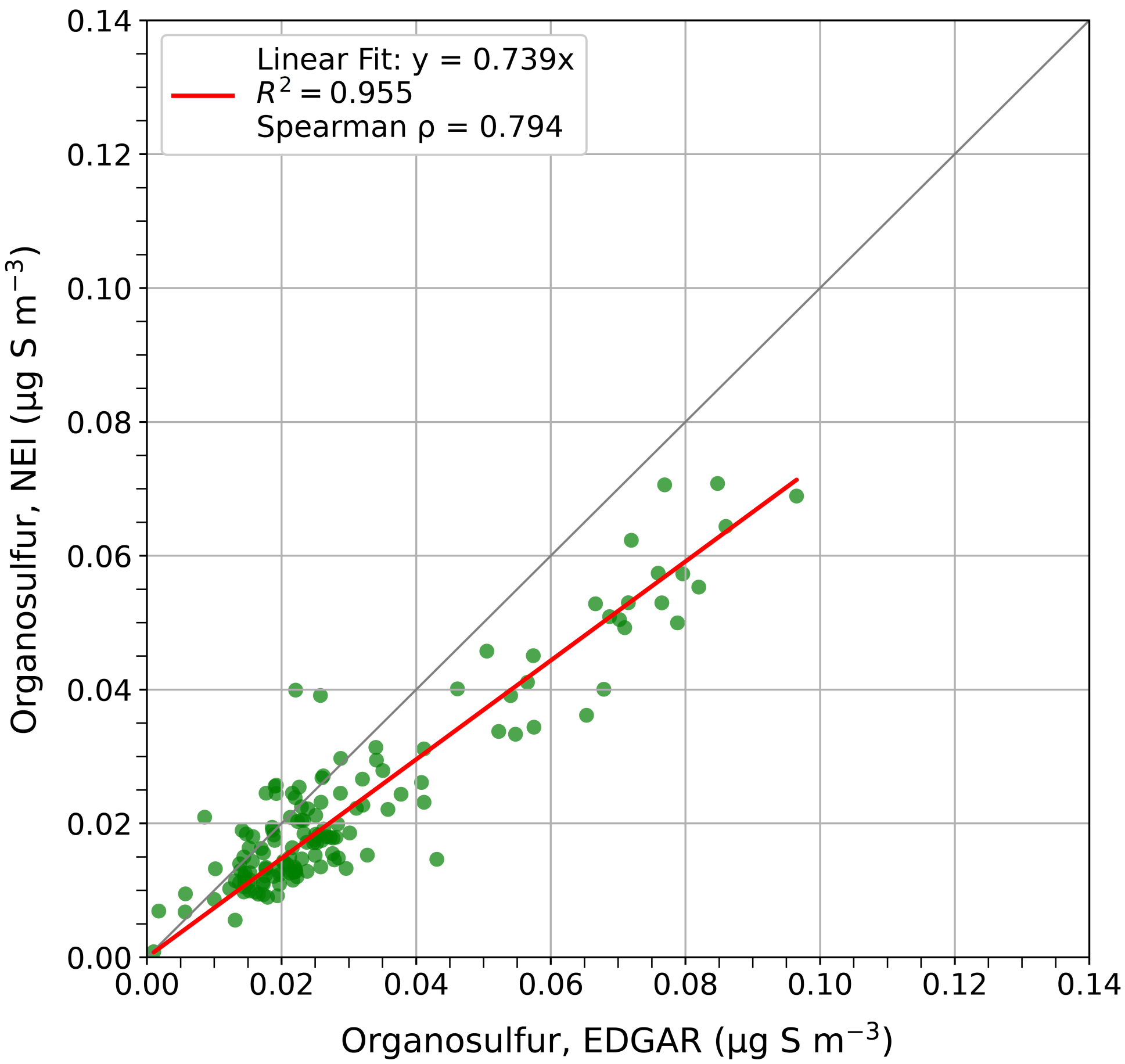

Figure S21. Scatter plot of $OrgS_{model}$ using anthropogenic emissions from NEI or EDGAR in July 2016, each point representing the modeled average concentration at an IMPROVE site.

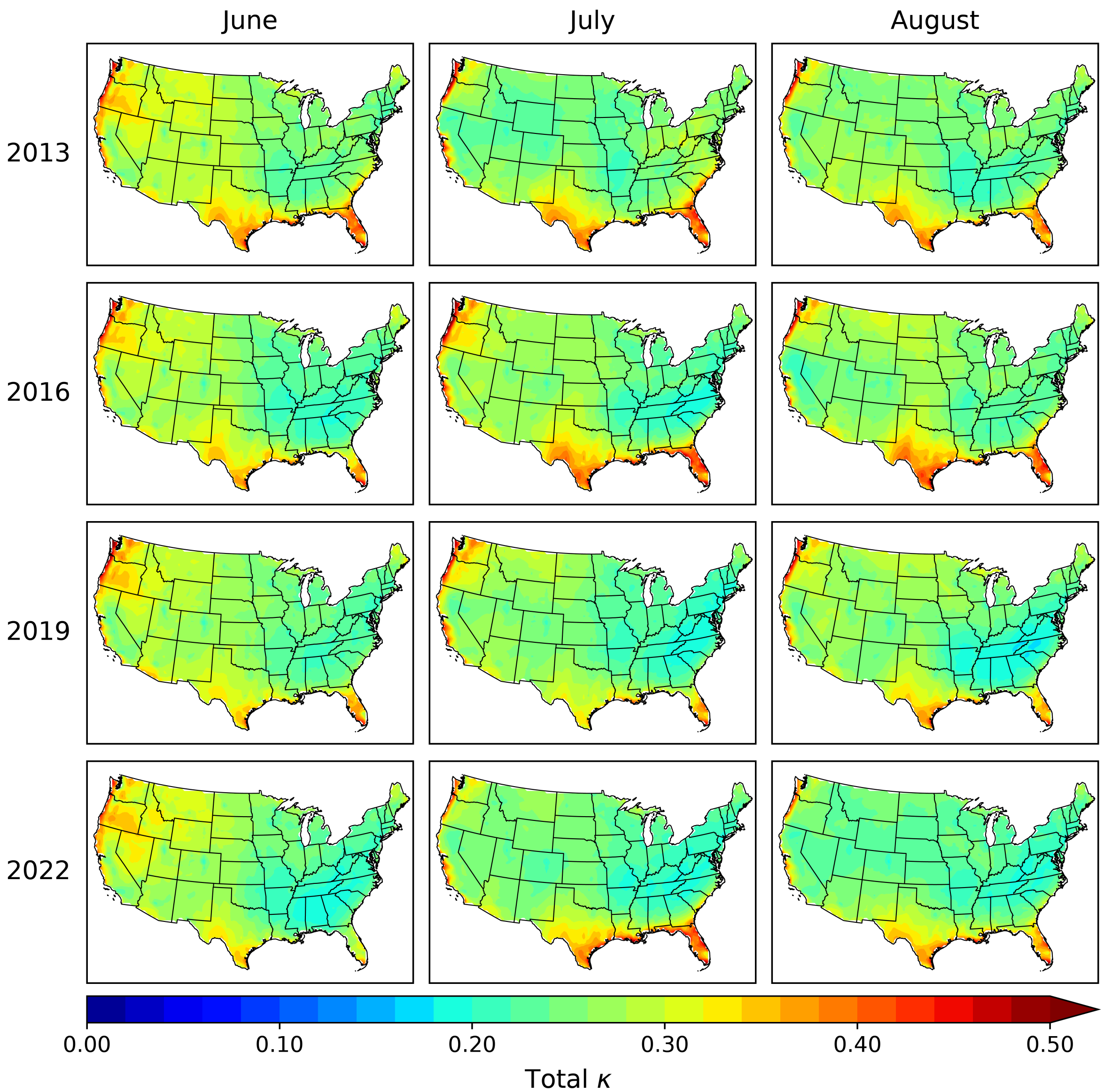

Figure S22. Estimations of total κ for each month, including the contributions of OSs.

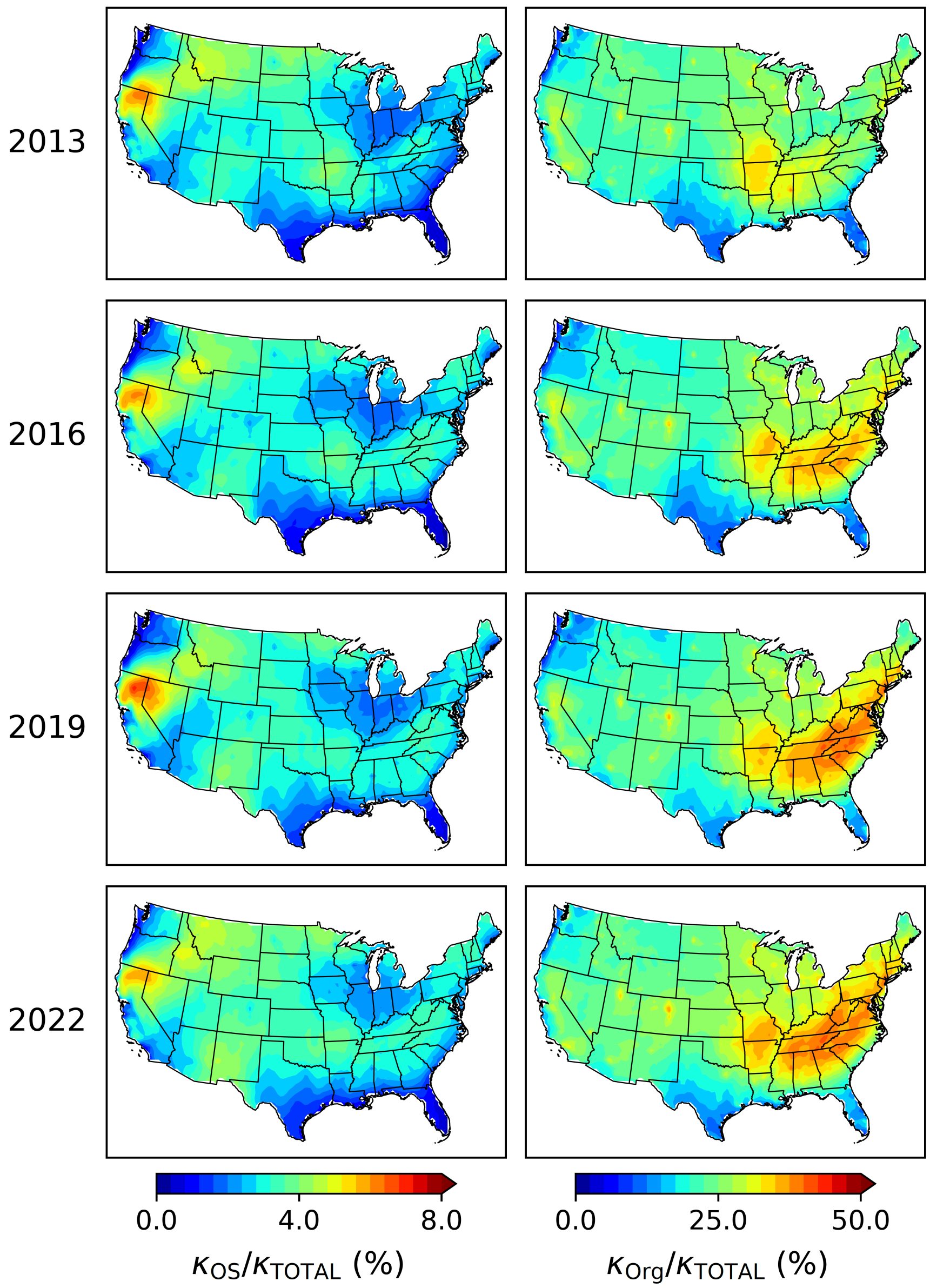

Figure S23. Fractional contributions of OSs and all organics to total *κ*.

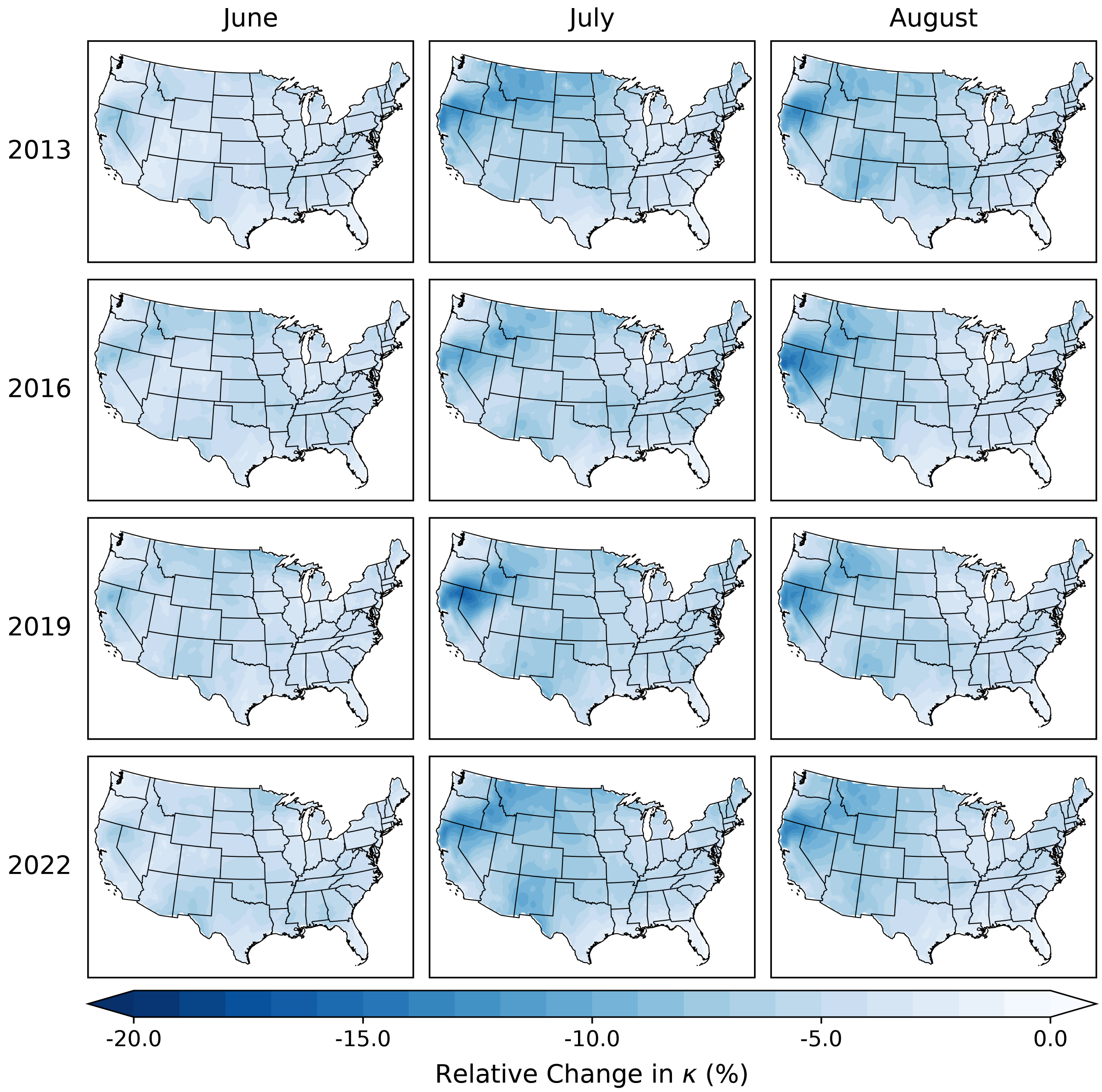

Figure S24. Relative change in total κ due to the inclusion of OSs for each month.

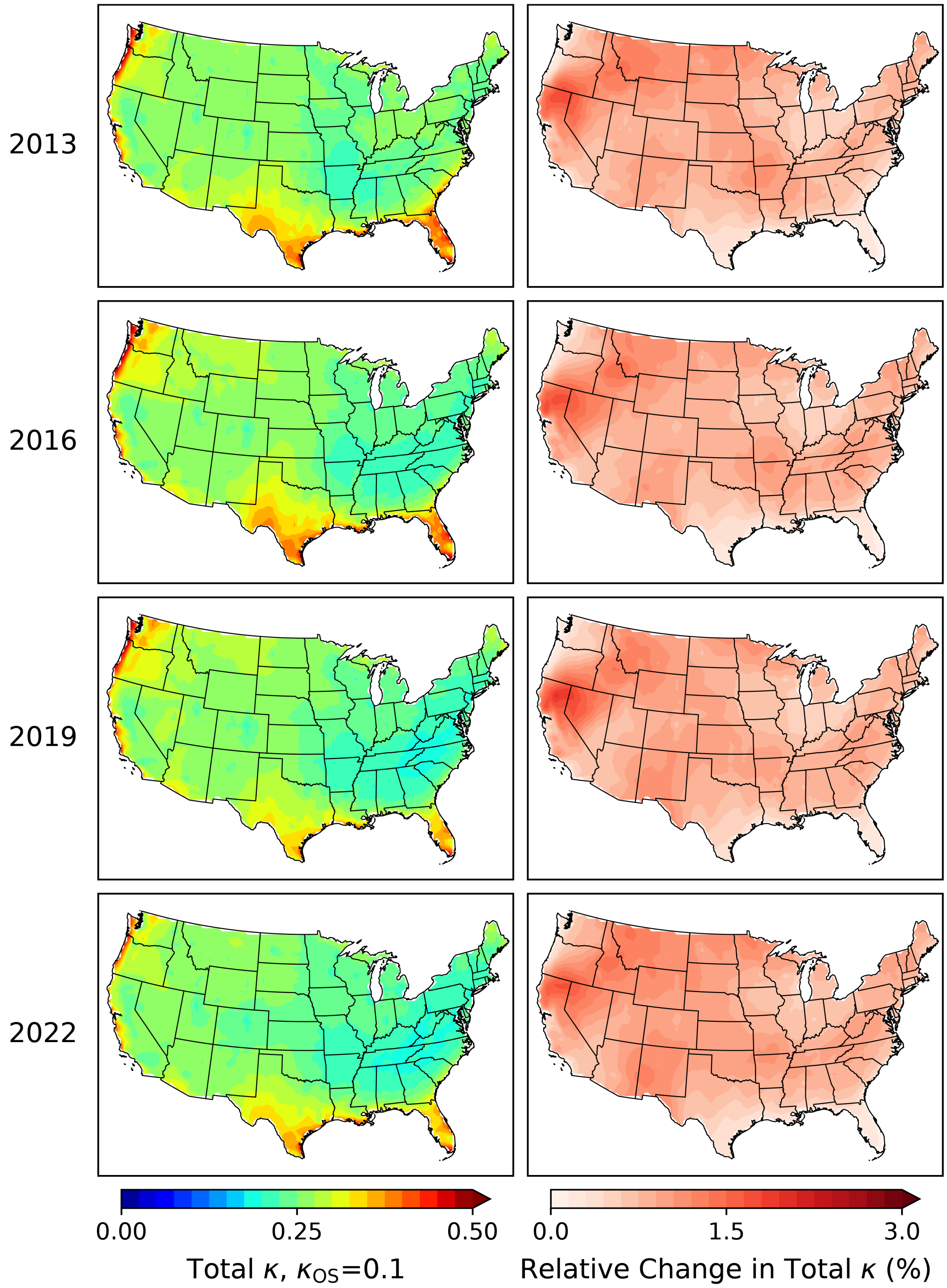

Figure S25. Impact of OSs κ value on total κ. Change in total κ considers an individual κ value of 0.139 for OSs instead of the κ value for lumped organics (0.1).

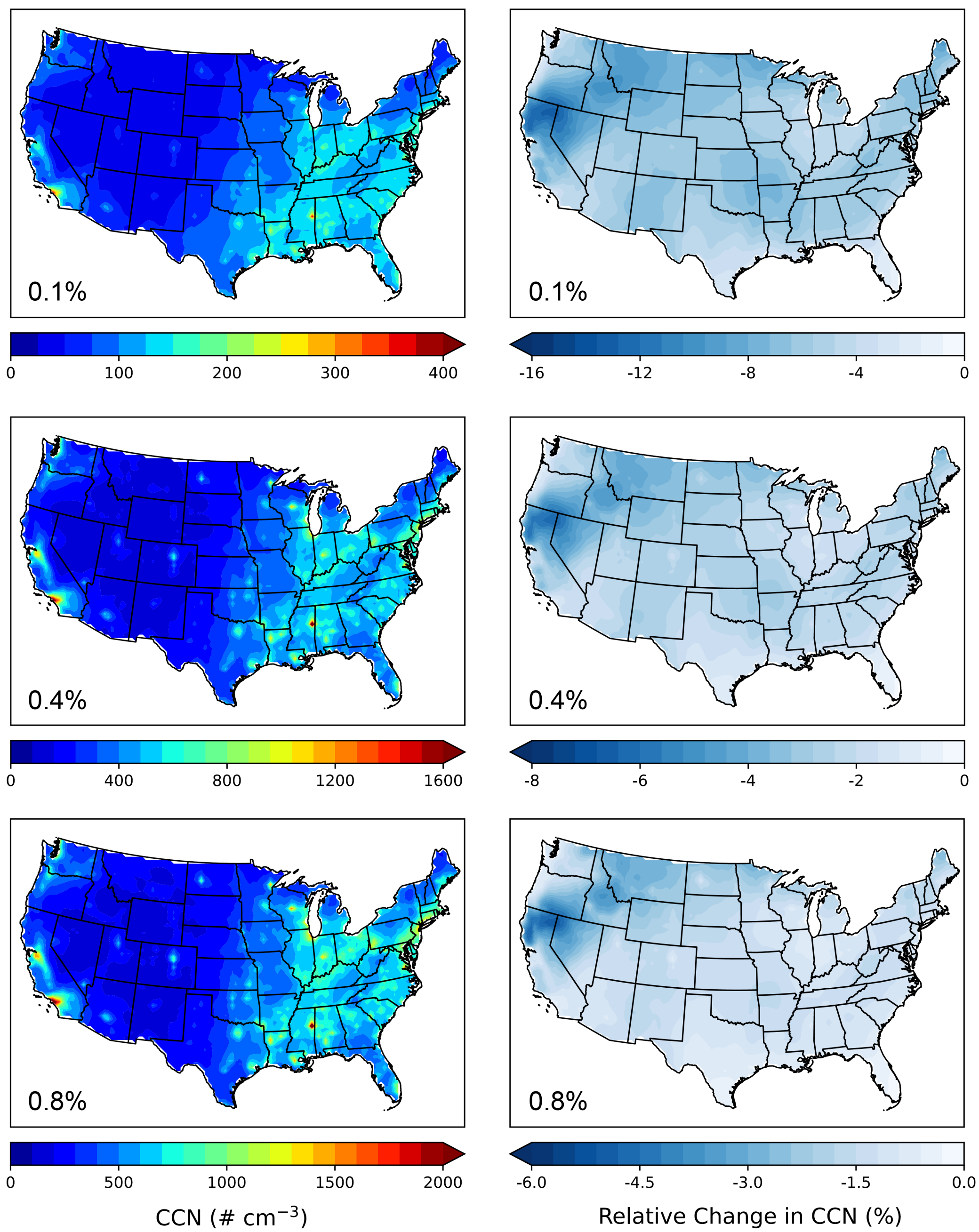

Figure S26. CCN number concentrations from fine particles and relative changes due to the inclusion of OSs in κ calculations. The supersaturation for each case is shown in the bottom-left corner of each map.

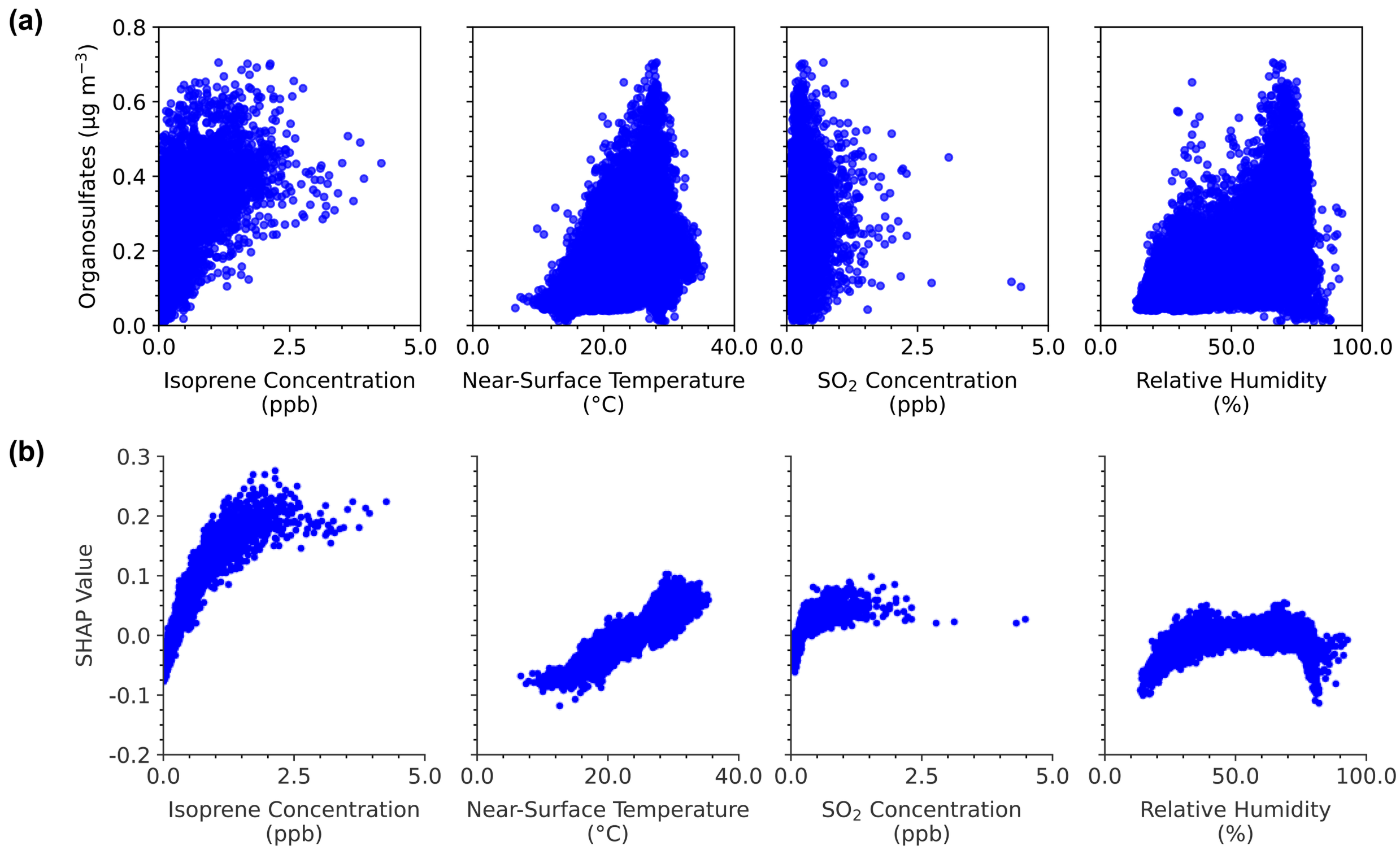

Figure S27. Relationships between (a) OS concentrations and (b) SHAP values for a subset of features used in the XGBoost model.

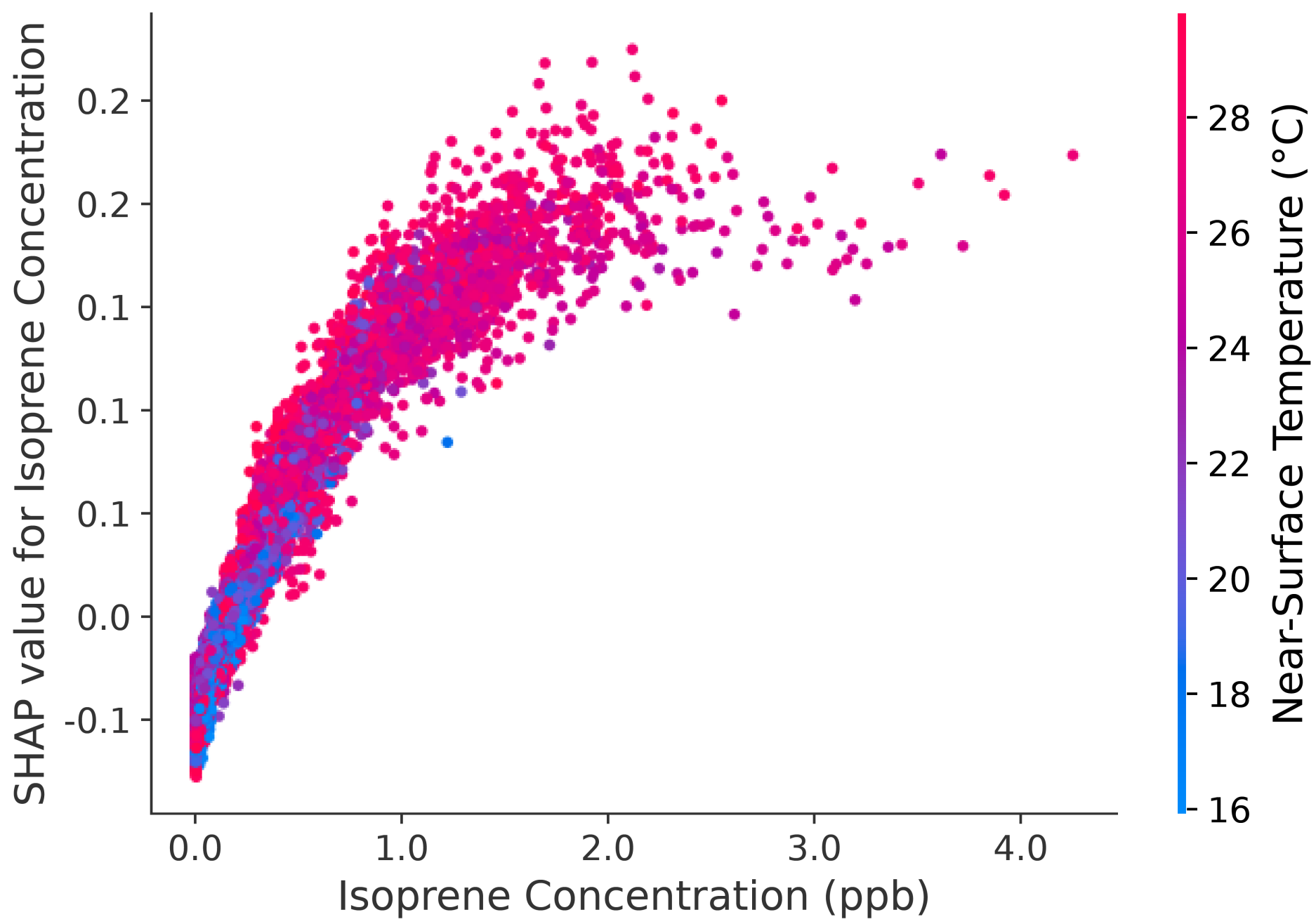

Figure S28. SHAP dependence plot for isoprene concentration and feature interactions with near-surface temperature based on predictions from the XGBoost model.

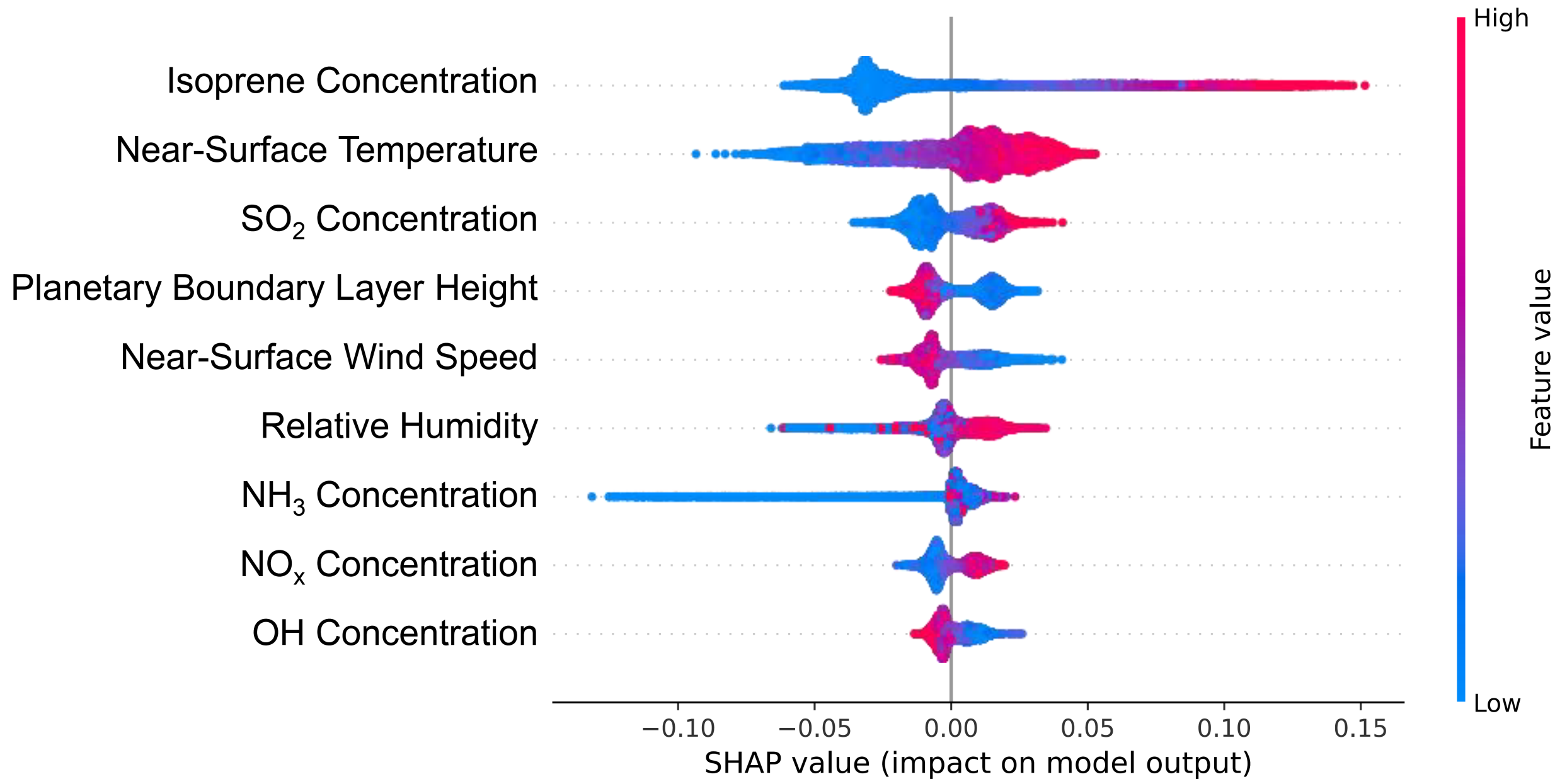

Figure S29. Contribution of predictive features to the modeled concentrations of OSs. A random forest model was trained with 3-fold cross-validation to assess the importance of features on the output concentrations of OSs.

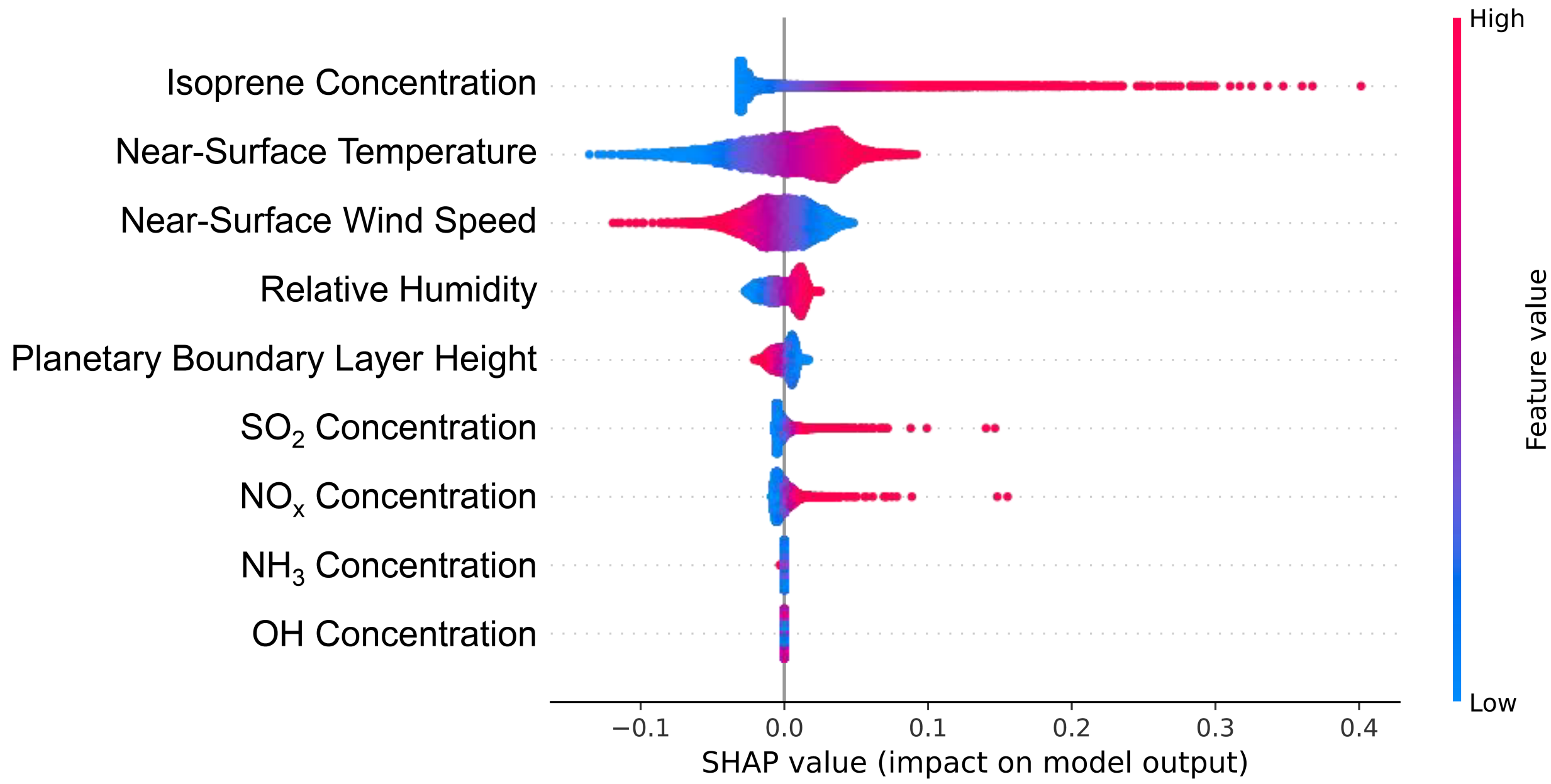

Figure S30. Same as Figure S29 but with a ridge regression model.

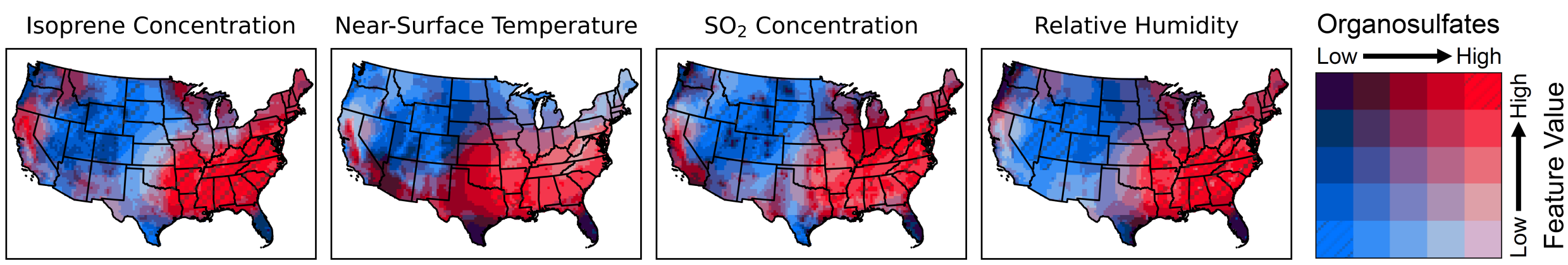

Figure S31. Bivariate maps of OS concentrations and primary predictive features. Maps display the intersection between features (isoprene concentration, near-surface temperature, SO_2 concentration, and relative humidity) and OS concentrations using a 5×5 color grid, with hatched areas indicating regions that exhibit strong interactions.

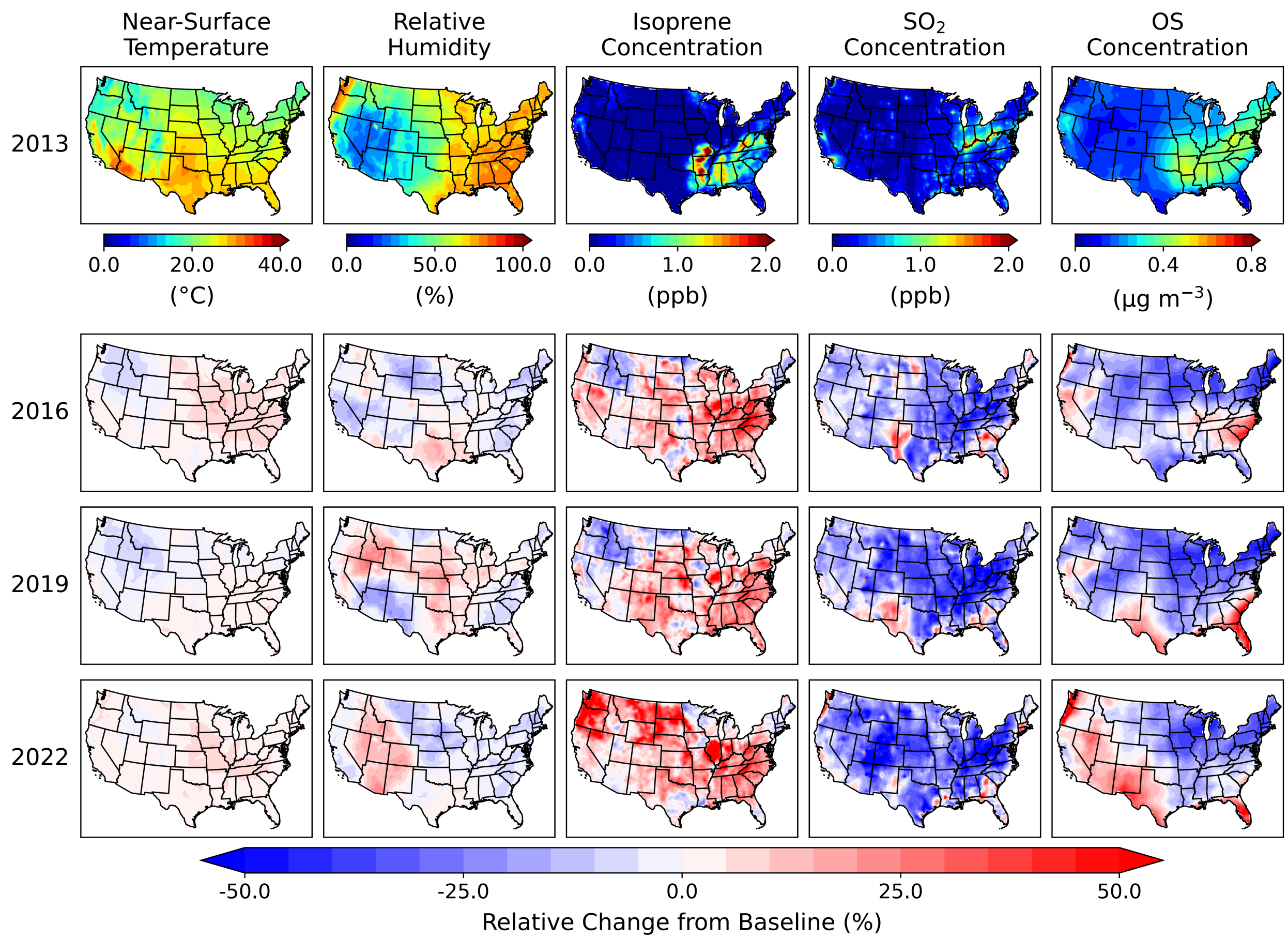

Figure S32. Summertime average and interannual change of selected predictive features of OSs. The selected features include isoprene and SO_2 gas-phase concentrations from the CMAQ output along with surface temperature and relative humidity from the meteorological data. The maps in the first row show the 2013 summertime average of each feature. The remaining maps show the change in values when compared to the 2013 summertime average. Modeled concentrations and interannual changes of the modeled OSs are included in the last column.

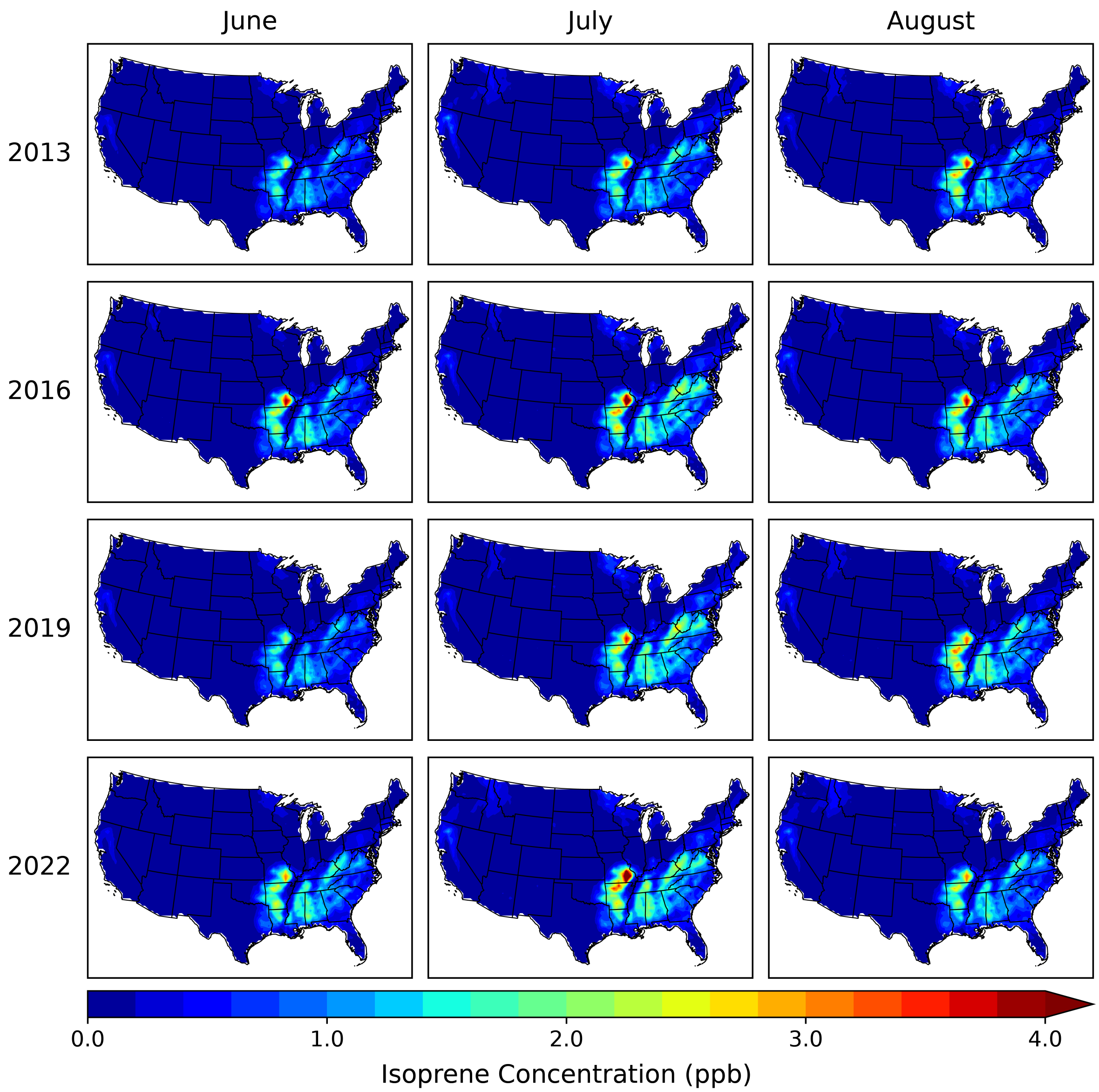

Figure S33. Monthly average isoprene concentrations.

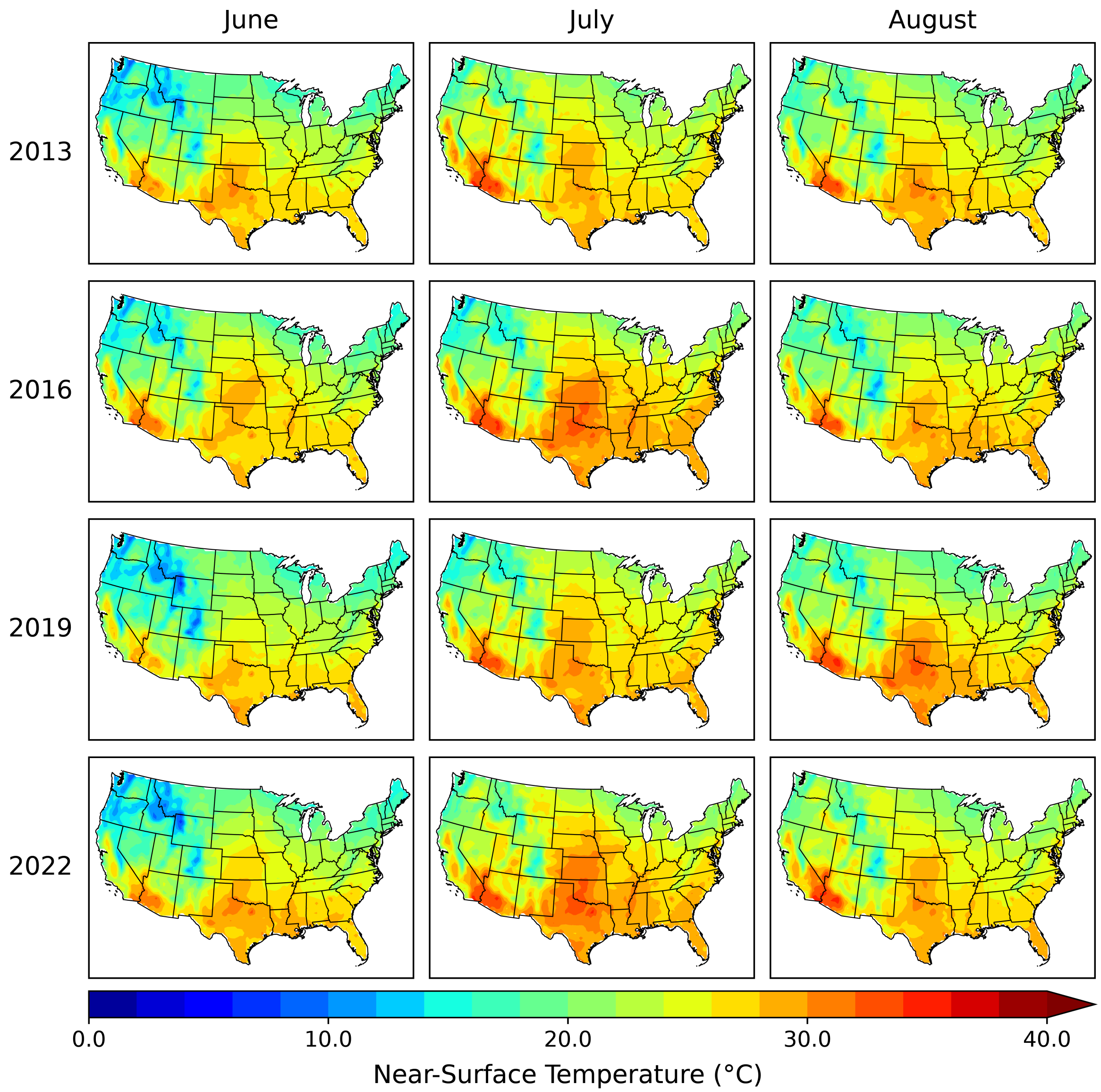

Figure S34. Monthly average near-surface temperature.

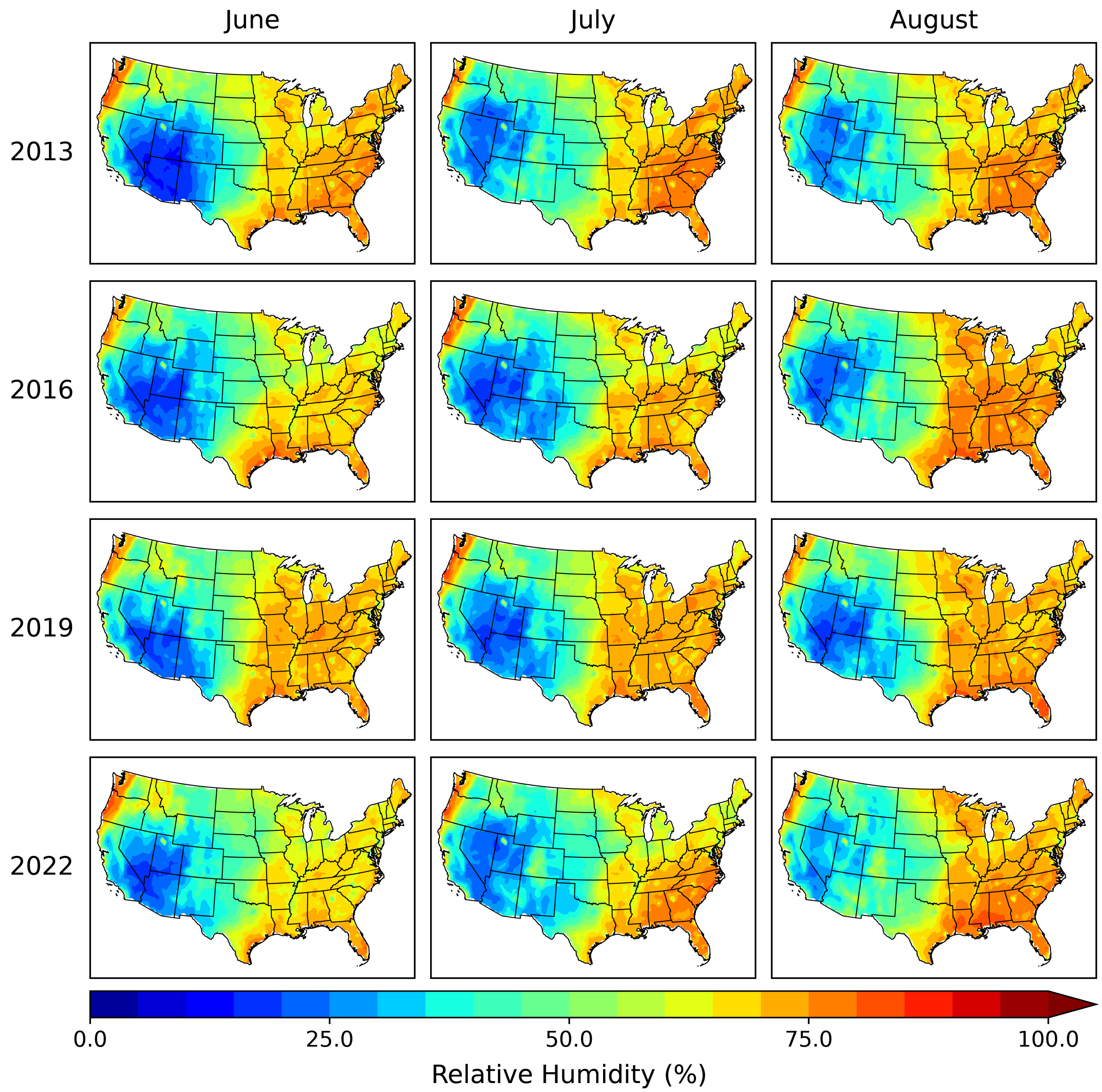

Figure S35. Monthly average relative humidity.

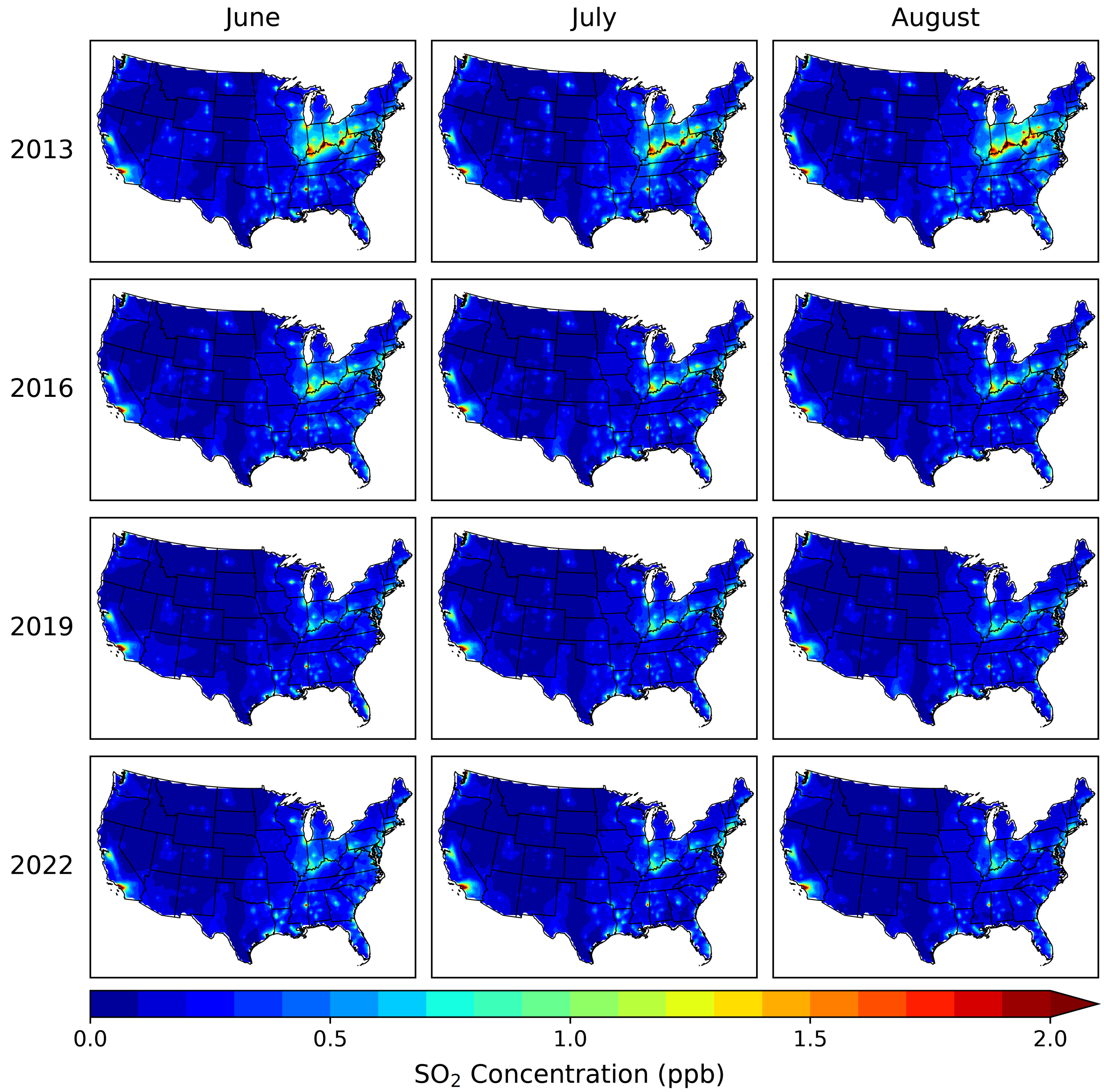

Figure S36. Monthly average SO_2 concentrations.

References

(1) Xie, Y.; Paulot, F.; Carter, W. P. L.; Nolte, C. G.; Luecken, D. J.; Hutzell, W. T.; Wennberg, P. O.; Cohen, R. C.; Pinder, R. W. Understanding the Impact of Recent Advances in Isoprene Photooxidation on Simulations of Regional Air Quality. *Atmospheric Chem. Phys.* **2013**, *13* (16), 8439–8455. https://doi.org/10.5194/acp-13-8439-2013

(2) Lin, Y.-H.; Zhang, Z.; Docherty, K. S.; Zhang, H.; Budisulistiorini, S. H.; Rubitschun, C. L.; Shaw, S. L.; Knipping, E. M.; Edgerton, E. S.; Kleindienst, T. E.; Gold, A.; Surratt, J. D. Isoprene Epoxydiols as Precursors to Secondary Organic Aerosol Formation: Acid-Catalyzed Reactive Uptake Studies with Authentic Compounds. *Environ. Sci. Technol.* **2012**, *46* (1), 250–258. https://doi.org/10.1021/es202554c

(3) Xu, R.; Chen, Y.; Ng, S. I. M.; Zhang, Z.; Gold, A.; Turpin, B. J.; Ault, A. P.; Surratt, J. D.; Chan, M. N. Formation of Inorganic Sulfate and Volatile Nonsulfated Products from Heterogeneous Hydroxyl Radical Oxidation of 2-Methyltetrol Sulfate Aerosols: Mechanisms and Atmospheric Implications. *Environ. Sci. Technol. Lett.* **2024**, *11* (9), 968–974. https://doi.org/10.1021/acs.estlett.4c00451

(4) Surratt, J. D.; Gómez-González, Y.; Chan, A. W. H.; Vermeylen, R.; Shahgholi, M.; Kleindienst, T. E.; Edney, E. O.; Offenberg, J. H.; Lewandowski, M.; Jaoui, M.; Maenhaut, W.; Claeys, M.; Flagan, R. C.; Seinfeld, J. H. Organosulfate Formation in Biogenic Secondary Organic Aerosol. *J. Phys. Chem. A* **2008**, *112* (36), 8345–8378. https://doi.org/10.1021/jp802310p

(5) Liu, X.; Zhang, B.; Ma, X.; Shi, Y.; Hou, K.; Qiu, J.; Shi, X.; Qi, S.; Yang, J. Reactive Evolution of HMML from Gas Phase Formation to Interface Transformation on Sulfuric Acid Aerosols: A Multiscale Computational Study. *J. Phys. Chem. A* **2025**, *129* (37), 8643–8652. https://doi.org/10.1021/acs.jpca.5c04295

(6) Lin, Y.-H.; Zhang, H.; Pye, H. O. T.; Zhang, Z.; Marth, W. J.; Park, S.; Arashiro, M.; Cui, T.; Budisulistiorini, S. H.; Sexton, K. G.; Vizuete, W.; Xie, Y.; Luecken, D. J.; Piletic, I. R.; Edney, E. O.; Bartolotti, L. J.; Gold, A.; Surratt, J. D. Epoxide as a Precursor to Secondary Organic Aerosol Formation from Isoprene Photooxidation in the Presence of

Nitrogen Oxides. *Proc. Natl. Acad. Sci.* **2013**, *110* (17), 6718–6723. https://doi.org/10.1073/pnas.1221150110

(7) Pye, H. O. T.; Pinder, R. W.; Piletic, I. R.; Xie, Y.; Capps, S. L.; Lin, Y.-H.; Surratt, J. D.; Zhang, Z.; Gold, A.; Luecken, D. J.; Hutzell, W. T.; Jaoui, M.; Offenberg, J. H.; Kleindienst, T. E.; Lewandowski, M.; Edney, E. O. Epoxide Pathways Improve Model Predictions of Isoprene Markers and Reveal Key Role of Acidity in Aerosol Formation. *Environ. Sci. Technol.* **2013**, *47* (19), 11056–11064. https://doi.org/10.1021/es402106h

(8) Zhu, S.; Zhang, J.; Li, L.; Zhou, M.; Qiao, L.; Wang, H.; Huang, D. D.; Wang, Q.; Jing, S.; Wu, Y.; Wang, S.; Chen, C.; Ying, Q.; Yu, J. Z. Aerosol Uptake Coefficients of Isoprene Epoxides: Determination and Parameter Estimation from Online Field Measurements of Organic Molecular Markers. *Environ. Sci. Technol.* **2025**, *59* (34), 18249–18258. https://doi.org/10.1021/acs.est.5c09046

(9) Schmedding, R.; Rasool, Q. Z.; Zhang, Y.; Pye, H. O. T.; Zhang, H.; Chen, Y.; Surratt, J. D.; Lopez-Hilfiker, F. D.; Thornton, J. A.; Goldstein, A. H.; Vizuete, W. Predicting Secondary Organic Aerosol Phase State and Viscosity and Its Effect on Multiphase Chemistry in a Regional-Scale Air Quality Model. *Atmospheric Chem. Phys.* **2020**, *20* (13), 8201–8225. https://doi.org/10.5194/acp-20-8201-2020

(10) Zhang, Y.; Chen, Y.; Lambe, A. T.; Olson, N. E.; Lei, Z.; Craig, R. L.; Zhang, Z.; Gold, A.; Onasch, T. B.; Jayne, J. T.; Worsnop, D. R.; Gaston, C. J.; Thornton, J. A.; Vizuete, W.; Ault, A. P.; Surratt, J. D. Effect of the Aerosol-Phase State on Secondary Organic Aerosol Formation from the Reactive Uptake of Isoprene-Derived Epoxydiols (IEPOX). *Environ. Sci. Technol. Lett.* **2018**, *5* (3), 167–174. https://doi.org/10.1021/acs.estlett.8b00044

(11) Eddingsaas, N. C.; VanderVelde, D. G.; Wennberg, P. O. Kinetics and Products of the Acid-Catalyzed Ring-Opening of Atmospherically Relevant Butyl Epoxy Alcohols. *J. Phys. Chem. A* **2010**, *114* (31), 8106–8113. https://doi.org/10.1021/jp103907c

(12) Petters, M. D.; Kreidenweis, S. M. A Single Parameter Representation of Hygroscopic Growth and Cloud Condensation Nucleus Activity. *Atmospheric Chem. Phys.* **2007**, *7* (8), 1961–1971. https://doi.org/10.5194/acp-7-1961-2007

(13) Chen, Y.; Dombek, T.; Hand, J.; Zhang, Z.; Gold, A.; Ault, A. P.; Levine, K. E.; Surratt, J. D. Seasonal Contribution of Isoprene-Derived Organosulfates to Total Water-Soluble Fine Particulate Organic Sulfur in the United States. *ACS Earth Space Chem.* **2021**, *5* (9), 2419–2432. https://doi.org/10.1021/acsearthspacechem.1c00102

(14) Hettiyadura, A. P. S.; Xu, L.; Jayarathne, T.; Skog, K.; Guo, H.; Weber, R. J.; Nenes, A.; Keutsch, F. N.; Ng, N. L.; Stone, E. A. Source Apportionment of Organic Carbon in Centreville, AL Using Organosulfates in Organic Tracer-Based Positive Matrix Factorization. *Atmos. Environ.* **2018**, *186*, 74–88. https://doi.org/10.1016/j.atmosenv.2018.05.007
